# International single-step SNPBLUP beef cattle evaluations for Limousin weaning weight

**DOI:** 10.1186/s12711-022-00748-0

**Published:** 2022-09-04

**Authors:** Renzo Bonifazi, Mario P. L. Calus, Jan ten Napel, Roel F. Veerkamp, Alexis Michenet, Simone Savoia, Andrew Cromie, Jérémie Vandenplas

**Affiliations:** 1 Animal Breeding and Genomics, Wageningen University & Research, P.O. Box 338, 6700 AH Wageningen, The Netherlands; 2 Interbull Centre—Department of Animal Breeding and Genetics, SLU-Box 7023, S-75007 Uppsala, Sweden; 3 Irish Cattle Breeding Federation, Link Road, Ballincollig, P31 D452 Co Cork Ireland

## Abstract

**Background:**

Compared to national evaluations, international collaboration projects further improve accuracies of estimated breeding values (EBV) by building larger reference populations or performing a joint evaluation using data (or proxy of them) from different countries. Genomic selection is increasingly adopted in beef cattle, but, to date, the benefits of including genomic information in international evaluations have not been explored. Our objective was to develop an international beef cattle single-step genomic evaluation and investigate its impact on the accuracy and bias of genomic evaluations compared to current pedigree-based evaluations.

**Methods:**

Weaning weight records were available for 331,593 animals from seven European countries. The pedigree included 519,740 animals. After imputation and quality control, 17,607 genotypes at a density of 57,899 single nucleotide polymorphisms (SNPs) from four countries were available. We implemented two international scenarios where countries were modelled as different correlated traits: an international genomic single-step SNP best linear unbiased prediction (SNPBLUP) evaluation (ssSNPBLUP_*INT*_) and an international pedigree-based BLUP evaluation (PBLUP_*INT*_). Two national scenarios were implemented for pedigree and genomic evaluations using only nationally submitted phenotypes and genotypes. Accuracies, level and dispersion bias of EBV of animals born from 2014 onwards, and increases in population accuracies were estimated using the linear regression method.

**Results:**

On average across countries, 39 and 17% of sires and maternal-grand-sires with recorded (grand-)offspring across two countries were genotyped. ssSNPBLUP_*INT*_ showed the highest accuracies of EBV and, compared to PBLUP_*INT*_, led to increases in population accuracy of 13.7% for direct EBV, and 25.8% for maternal EBV, on average across countries. Increases in population accuracies when moving from national scenarios to ssSNPBLUP_*INT*_ were observed for all countries. Overall, ssSNPBLUP_*INT*_ level and dispersion bias remained similar or slightly reduced compared to PBLUP_*INT*_ and national scenarios.

**Conclusions:**

International single-step SNPBLUP evaluations are feasible and lead to higher population accuracies for both large and small countries compared to current international pedigree-based evaluations and national evaluations. These results are likely related to the larger multi-country reference population and the inclusion of phenotypes from relatives recorded in other countries via single-step international evaluations. The proposed international single-step approach can be applied to other traits and breeds.

**Supplementary Information:**

The online version contains supplementary material available at 10.1186/s12711-022-00748-0.

## Background

In livestock species, genomic selection [1] has become increasingly important for driving selection decisions of both economically and socially relevant traits [2–4]. In animal breeding programs, the inclusion of genomic data in addition to conventional sources of information (pedigree and phenotypes) leads to an increase in prediction accuracy of estimated breeding values (EBV) and to reduced generation intervals which, in turn, allows to achieve higher genetic gains [5]. National genomic evaluations make use of phenotypic, genomic and pedigree information either based on multi-step approaches [6] or single-step approaches [7, 8]. Although genotyping is becoming cheaper and the availability of individual genomic data is increasing [3], accurate genomic predictions often require large and representative reference populations [2, 9–11], which can be expensive and time-consuming to build and maintain, especially for difficult-to-measure traits [12, 13]. Moreover, with small livestock populations, building a reference population using only national resources can be challenging or even unfeasible. For such small national populations, a combined international genomic evaluation is appealing, especially when genomic predictions are performed within-breed [14, 15].

In cattle, the aim of international collaborations projects is to pull together genomic data from different countries and build large reference populations within the same breed [16]. These projects allow to either (1) share genotypes and breeding values as pseudo-phenotypes among national breeding organizations and, in turn, enlarge existing national reference populations, or (2) perform an international genomic evaluation for which raw national data or a proxy of them (e.g. de-regressed proofs [17]) are used. Examples of international collaboration projects in dairy cattle are the “North America Consortium” [18], “EuroGenomics” [19], and “InterGenomics” [20].

Genomic selection has also been increasingly adopted in beef cattle national evaluations [21–24], however, compared to dairy cattle [22, 25] some additional difficulties exist. In particular, the lower use of artificial insemination (which results in lower connectedness between herds and smaller sire families) and the lower systematic recording of phenotypes compared to dairy breeding programs contribute to smaller benefits of genomic selection in beef cattle. These difficulties also make international evaluations more challenging in beef cattle compared to dairy, especially due to low pedigree connectedness between countries [22, 26]. International genomic evaluations may contribute to increase connectedness among countries by using genomic data along with pedigree and phenotypic data [27]. Moreover, genomic data would help to combine (small) national reference populations into an international one and, in turn, result in an increase in the accuracies of genetic evaluations. However, to date, the current international beef cattle evaluations led by Interbeef [28] do not yet consider genomic information.

When properly parametrized, it is expected that the optimal approach for international genomic evaluations would be to implement a single-step evaluation because it allows combining phenotypic, genomic and pedigree national information simultaneously [29], using raw national phenotypes and genotypes without the need to approximate them. Moreover, such a model would need to be easily scalable to efficiently handle a large number of traits and a large amount of (genomic) data. To date, the feasibility and the benefits of joint single-step beef cattle international evaluations have not been explored. Therefore, the aim of our study was to develop a joint international single-step genomic evaluation for beef cattle using Limousin weaning weight data, and to investigate its benefits for both the increase in accuracies of EBV and genetic connectedness among countries over the current pedigree-based Interbeef evaluations. Moreover, we evaluated whether moving towards international single-step genomic evaluations would affect level and dispersion bias of EBV compared to both current international pedigree-based evaluations and national evaluations.

## Methods

Hereafter, we first describe the available data and its preparations steps, followed by the implemented scenarios and a description of the international models used. Finally, we describe the assessment of connections among countries and the validation methodology implemented.

### Phenotypes

In total, 333,333 Limousin male and female age-adjusted weaning weight (AWW) phenotypes were available. Phenotypes were available from five Limousin populations, representing seven European countries joining the 2020 January Interbeef evaluation. These countries were: Czech Republic (CZE), Denmark, Finland and Sweden (DFS, modelled as one population), Ireland (IRL), Germany (DEU), and Switzerland (CHE). Weaning weight was age-adjusted to 210 days in CZE and to 200 days in the remaining countries. For further details on data recording and adjustment factors at the national level see Additional file 1 Table S3 in Bonifazi et al. [30], and Interbeef National Genetic Evaluations forms [31]. Phenotypes above or below three phenotypic standard deviations from the phenotypic mean of each population-sex combination were discarded to remove possible outliers. In total, 331,593 individual phenotypic records remained and were distributed across 19,051 herds. The number of phenotypes available in each population is reported in Table 1. DEU represented the largest population with 35% of the observations, followed by DFS (29%), IRL (21%), CHE (11%), and CZE (4%). Recorded animals were born between 1975 and 2019. Descriptive statistics per population of the available phenotypes are in Additional file 1: Table S1. Hereafter, although the DFS population is composed of more than one country, for simplicity, we will refer to populations as “countries”.

**Table 1:** Summary of available data per country

Country	AWW	AWW %	Herds	Year of birth (min–max)	Genotypes	% genotypes	Genotypes with phenotypes^a^	% genotypes with phenotypes^a^
CZE	13,892	4	172	1991–2019	1625	9	1207	74
DFS	96,671	29	9548	1980–2019	–	–	–	–
IRL	68,086	21	8218	1975–2019	11,300	64	5237	46
DEU	117,249	35	866	1981–2019	742	4	640^b^	86
CHE	35,695	11	247	1992–2018	3940	22	3516	89
Total	331,593	100	19,051	1975–2019	17,607	100	10,600	60

*CZE* Czech Republic; *DFS* Denmark, Finland and Sweden; *IRL* Ireland; *DEU* Germany; *CHE* Switzerland; *AWW* Age-adjusted weaning weight

^a^Genotypes with an associated phenotype in the country

^b^49 and 1 genotypes with associated phenotypes in DEU were sent from CHE and IRL, respectively

### Pedigree

The pedigree was extracted from the Interbeef international pedigree database and the following quality controls were performed. The pedigree was checked for absence of pedigree loops (an animal being its own ancestor), duplicates, and conflicts between the sex reported in the international identification number and its sex as a parent (e.g. a female reported in the pedigree as a sire). Finally, using the RelaX2 software v1.73 [32], the checked pedigree was pruned to include animals with phenotypes, genotypes, or both, and all their ancestors, without any limit on the number of generations retained. The final pedigree included 519,740 animals, born between 1927 and 2019, with a maximum depth of 17 generations.

### Genotypes

Genotypes were available for 17,733 animals from four countries: CZE, DEU, IRL, and CHE. Genotypes were available from single nucleotide polymorphism (SNP) panels with different SNP densities: 467 DEU animals at 41,913 SNPs (42K), 11,354 IRL animals at 52,690 SNPs (52K), 1004 CZE animals at 53,218 SNPs (53K), 278 DEU animals at 54,609 SNPs (55K), and 648 CZE and 3982 CHE animals at 139,480 SNPs (139K). SNPs were coded as 0 and 2 for the two homozygotes, and as 1 for the heterozygote. Genotypes originating from different sources were merged using the unambiguous A/B Illumina allele coding [33].

#### Imputation to a selected panel of SNPs

Genotypes sent by various countries were imputed to a selected panel of SNPs as described in the following sections.

##### Pre-imputation genomic data edits

Only autosomal SNPs with a known name and chromosome position for the *Bos taurus* UMD 3.1 bovine genome assembly [34] were retained from each SNP panel. Duplicated SNPs that were at the same physical position within a genotyping SNP panel were discarded. The selected panel of SNPs consisted of 147,511 autosomal SNPs. Before imputation, 22 genotypes (8 sent by CHE and 14 sent by IRL) were removed due to duplication, and the following edits were applied to the available genotypes.

1. SNPs in common with the selected panel were retained from each country genotype; the remaining SNPs were discarded. Table 2 reports the number of retained SNPs from each panel, together with the number of SNPs in common between panels.
2. SNPs with too many Mendelian conflicts were removed. A SNP was removed from the selected panel if the number of parent–offspring genotype conflicts exceeded 1% of the parent–offspring pairs that both have their genotype called for this SNP. In total, 1411 SNPs were removed.
3. Detection of parent–offspring conflicts. A parent–offspring conflict was detected when the number of conflicting autosomal SNPs between the parent–offspring pair exceeded 1% of the number of SNPs shared between the SNP panel used for the parent and the offspring (as in Table 2). For each parent–offspring conflict detected, the pedigree link between the parent and the offspring was removed by setting the offspring’s parent to missing. Nineteen parent–offspring pedigree links were removed.

After the above edits, 146,100 SNPs and 17,711 genotypes (out of the initial 17,733) were used for imputation.

**Table 2:** Number of autosomal SNPs retained in each panel (diagonal), across panels (off-diagonal), and between each panel and the selected panel of SNP (i.e. 147,511 SNPs) (diagonal)

Panel	42K	52K	53K	55K	139K
42K	40,367				
52K	36,519	44,181			
53K	39,907	40,283	51,250		
55K	39,465	39,779	48,744	52,352	
139K	36,270	37,665	40,550	39,990	131,584

42K = 41,913 SNPs, 52K = 52,690 SNPs, 53K = 53,218 SNPs, 55K = 54,609 SNPs, 139K = 139,480 SNPs

##### Imputation and quality control

All the genotypes were imputed to the selected panel using Findhap v3 [35] (applied settings are in Additional file 2: File S1). After imputation, the following quality controls were performed using Plink v1.9 [36]: (1) call rates per SNP across animals ≥ 95%; (2) SNPs with a *p-value* for Hardy–Weinberg equilibrium Chi-square test higher than 10^–15^; (3) SNPs with a minor allele frequency higher than 0.01; and (4) call rates per animal across SNPs ≥ 90%. SNPs and genotypes that did not match these criteria were removed and thus 17,688 genotypes and 57,899 SNPs remained. The percentage of autosomal SNPs retained after quality control relative to the original panels were 89%, 94%, 77%, 74%, and 40%, for the 42K, 52K, 53K, 55K, and 139K panel, respectively. In addition, genotypes with less than 87.5% pedigree-based breed composition for the Limousin breed were removed (32 genotypes excluded), and genotypes with pedigree incompatibilities (observed from plotting genomic against pedigree-based relationships; see Additional file 3: Fig. S1) were also removed (41 genotypes excluded). Finally, genotypes of animals without phenotypic records, progeny and known parents were removed (8 genotypes excluded). The final number of genotypes was 17,607 and their distribution per country is in Table 1; the majority were from IRL (64% of the total), followed by CHE (22%), CZE (9%) and DEU (4%). Principal component analysis (PCA) was performed on the genomic relationship matrix of all genotypes to further investigate connectedness between countries for genotyped animals. The program calc_grm [37] was used to build the genomic relationship matrix following VanRaden’s method 2 [6] and to perform the PCA following Patterson et al. [38].

### Scenarios

To investigate the benefits of including genomic information in international evaluations compared to the current international pedigree-based evaluation, we implemented the following two scenarios, hereafter referred to as “international scenarios”.

- Scenario PBLUP_*INT*_: international pedigree-based best linear unbiased prediction (BLUP) evaluation (as described below) using all available phenotypes. This scenario represents the current Interbeef international evaluations.
- Scenario ssSNPBLUP_*INT*_: international single-step SNPBLUP evaluation (as described below) using all available phenotypes and genotypes.

In both international scenarios, the complete international pedigree was used.

To investigate the benefits of international evaluations compared to national evaluations, we also implemented the following two scenarios, hereafter referred to as “national scenarios”, which aim at representing national single trait evaluations.

- Scenario PBLUP_*NAT*_: national pedigree-based BLUP evaluation, performed separately for each country using only national submitted phenotypes (as reported in Table 1).
- Scenario ssSNPBLUP_*NAT*_: national single-step SNPBLUP evaluation, performed separately for each country using only national submitted phenotypes and genotypes (as reported in Table 1). DFS was excluded for this scenario as no genotypes were available.

In both national scenarios, the complete international pedigree was used for the estimation of both pedigree and single-step EBV. In each national scenario, the EBV of animals that appear in a pseudo-national pedigree were used. Pseudo-national pedigrees were obtained by pruning the international pedigree to include all national animals with phenotypes, genotypes, or both, and all their ancestors, without any limit on the number of generations retained. National scenarios used the same within-country variance components as the international scenarios. Table 3 presents a summary of the sources of information included in both international and national scenarios and Table 4 reports the number of phenotypes, genotypes and size of the pedigree in each scenario.

**Table 3:** Sources of information included (•) in implemented scenarios

Sources of information	National scenarios		International scenarios	
	PBLUP_*NAT*_	ssSNPBLUP_*NAT*_	PBLUP_*INT*_	ssSNPBLUP_*INT*_
Within-country national pedigree^a^	•	•	•	•
Within-country national phenotypes	•	•	•	•
Within-country national genotypes		•		•
Across-country international pedigree			•	•
Across-country international phenotypes			•	•
Across-country international genotypes				•

*PBLUP*_*NAT*_ pedigree-based BLUP national, *ssSNPBLUP*_*NAT*_ single-step SNP-BLUP national, *PBLUP*_*INT*_ pedigree-based BLUP international, *ssSNPBLUP*_*INT*_ single-step SNP-BLUP international

^a^Within-country national pedigree is a pseudo-national pedigree obtained by pruning the international pedigree to include all national animals with phenotypes, genotypes, or both, and all their ancestors, without any limit on the number of generations retained

**Table 4:** Number of phenotypes, genotypes, and size of the pedigree in whole and partial evaluations of international and national scenarios, and number of animals in the focal group for each country

Evaluation		Scenarios					
		International	National				
			CZE	DFS	IRL	DEU	CHE
*Whole*	Pedigree	519,740	44,130	125,743	186,080	165,318	67,567
	Genotypes	17,607	1625	–	11,300	742	3940
	Phenotypes	331,593	13,892	96,671	68,086	117,249	35,695
*Partial*	Phenotypes	243,109	7104	78,730	49,579	82,876	24,820
Focal group		–	1057	–	3869	407	1191

*Partial*: is the same as *whole* evaluation but with phenotypes of animals born from 2014 onwards excluded

International scenarios: *PBLUP*_*INT*_ pedigree-based BLUP international and *ssSNPBLUP*_*INT*_ single-step SNP-BLUP international

National scenarios: *PBLUP*_*NAT*_ pedigree-based BLUP national and *ssSNPBLUP*_*NAT*_ single-step SNP-BLUP national

Focal group: animals with phenotypes and genotypes born from 2014 onwards

*CZE* Czech Republic; *DFS* Denmark, Finland and Sweden; *IRL* Ireland; *DEU* Germany; *CHE* Switzerland

### Models

#### International pedigree-based BLUP

The current Interbeef model for breeding value estimation without genomic information is the AMACI model (Animal Model accounting for Across-Country Interaction) [39], which accounts for country-specific fixed and random effects by fitting the national model of each country. The AMACI model is a multi-trait animal model with maternal effects, in which each country is modelled as a different trait:

$${\mathbf{y}}_{i}={\mathbf{X}}_{i}{\mathbf{b}}_{i}+{\mathbf{C}}_{i}{\mathbf{r}}_{i}+{\mathbf{Z}}_{i}{\mathbf{u}}_{i}+{\mathbf{W}}_{i}{\mathbf{m}}_{i}+\boldsymbol{ }{\mathbf{P}}_{i}{\mathbf{p}}_{i}+\boldsymbol{ }{\mathbf{e}}_{i},$$

where ${\mathbf{y}}_{i}$ is the vector of observations for country $i$; ${\mathbf{b}}_{i}$ is the vector of fixed effects for country $i$; ${\mathbf{r}}_{i}$ is the vector of random environmental effects for country $i$; ${\mathbf{u}}_{i}$ is the vector of random additive genetic (direct) effects for country $i$; ${\mathbf{m}}_{i}$ is the vector of random maternal additive genetic effects for country $i$; ${\mathbf{p}}_{i}$ is the vector of random maternal permanent environmental effects (provided by the dam) for country $i$; ${\mathbf{e}}_{i}$ is the vector of random residual effects for country $i$. ${\mathbf{X}}_{i}$ and ${\mathbf{C}}_{i}$ are incidence matrices linking records to fixed, and random environmental effects, respectively. ${\mathbf{Z}}_{i}$, ${\mathbf{W}}_{i}$, and ${\mathbf{P}}_{i}$ are incidence matrices linking records to the animal, maternal genetic and maternal permanent environmental effects, respectively. National fixed and random effects for each country are in Additional file 1: Table S2. Random environmental effects were modelled for three countries: CZE (herd-year-season), DEU (herd-year), and CHE (herd-year). Following the national model, the maternal permanent environmental effect was not fitted for the DEU population. It was assumed that:

$$Var\left[ {\begin{array}{*{20}c} {\mathbf{u}} \\ {\mathbf{m}} \\ \end{array} } \right] = {\mathbf{G}} \otimes {\mathbf{A}} = \left[ {\begin{array}{*{20}c} {{\mathbf{G}}_{{d,d}} }\quad{\mathbf{G}}_{{d,m}} \\ {{\mathbf{G}}_{{m,d}} }\quad{\mathbf{G}}_{{m,m}} \\ \end{array} } \right] \otimes {\mathbf{A}},$$

where $\mathbf{u}$ is the vector of random direct additive genetic effects for all countries; $\mathbf{m}$ is the vector of random maternal additive genetic effects for all countries; $\mathbf{G}$ is the across-country genetic co-variance matrix of order 10 × 10, in which ${\mathbf{G}}_{d,d}$ is the across-country direct ($d$) additive genetic co-variance matrix; ${\mathbf{G}}_{m,m}$ is the across-country maternal ($m$) additive genetic co-variance matrix; and ${\mathbf{G}}_{d,m}$ (${\mathbf{G}}_{m,d})$ contains additive genetic covariances between direct and maternal effect within countries (diagonal elements) and additive genetic co-variances between direct and maternal effect across countries (off-diagonal elements); $\mathbf{A}$ is the numerator relationship matrix; $\otimes$ indicates the Kronecker product. Random environmental effects, random maternal permanent environmental effects, and residuals were fitted using block-diagonal variance matrices.

The genetic co-variance matrix with additive direct and maternal genetic effects ($\mathbf{G}$) was built as:

$$\mathbf{G} = \mathbf{S} {\varvec{\Phi}} \mathbf{S},$$

where, $\mathbf{S}$ is the diagonal matrix with national genetic standard deviations for direct and maternal genetic effects, and ${\varvec{\Phi}}$ is the across-country estimated genetic correlation matrix (of order 10 × 10 with diagonal values of 1). The across-country ${\varvec{\Phi}}$ matrix was constructed by combining the genetic correlations between countries previously estimated by Interbeef using a series of bi-variate analyses. The resulting combined across-country ${\varvec{\Phi}}$ matrix was not positive definite and a bending procedure was applied using the R package “mbend” [40] (method “hj” [41], unweighted, with a threshold value of 10^–4^). The final across-country genetic correlation matrix ${\varvec{\Phi}}$ and $\mathbf{G}$ co-variance matrix are reported in Additional file 1: Table S3. Both within-country genetic and environmental variances were the same as those used in the national genetic evaluations of participating countries and are in Additional file 1: Table S4. Interbeef uses this procedure to compute the genetic co-variance matrix under the assumption that the national estimates of genetic variances are more accurate (e.g. when not all national data are submitted for international evaluations) [42]. Possible differences in trait and model definition between countries, as well as genotype-by-environment interactions, are accounted for in the AMACI model by modelling each country as a different correlated trait and with genetic correlations between countries lower than 1 [30, 43].

#### International single-step SNPBLUP

Genomic data was included in the AMACI model using a single-step SNP BLUP (ssSNPBLUP) approach as proposed by Liu et al. [44] and later applied by Vandenplas et al. [45] to multi-trait models with maternal genetic effects. Following Vandenplas et al. [45], observed allele frequencies were used to center the SNP genotypes. The estimated co-variance components used for the ssSNPBLUP evaluation were the same as the estimated co-variance components used for the pedigree-based BLUP evaluation. The proportion of variance (due to additive genetic effects) considered as due to residual polygenetic effects was assumed to be 5%. For further details on the ssSNPBLUP evaluation applied to a multi-trait model with a maternal effect, see Vandenplas et al. [45].

The compatibility between pedigree and genomic information was guaranteed by fitting two $\mathbf{J}$ covariates (corresponding to the additive and maternal genetic effects) as fixed effects in the model [46]. Such compatibility is required to account for allele frequencies being computed from the observed genotypes rather than from the unknown base population [46, 47]. In short, $\mathbf{J}$ covariates model the genetic level of genotyped animals ensuring the compatibility of genomic information with that of animals without genotypes in single-step approaches. $\mathbf{J}$ covariates are computed as follows [48]. First, entries of $\mathbf{J}$ corresponding to genotyped animals ($g$) are set to − 1, i.e. ${\mathbf{J}}_{g}={\mathbf{-1}}$. Second, covariate values for non-genotyped ancestors of genotyped individuals ($anc$) are computed as ${\mathbf{J}}_{anc}={\mathbf{A}}_{anc,g}{\left({\mathbf{A}}_{g,g}\right)}^{-1}{\mathbf{J}}_{g}$, where ${\mathbf{A}}_{anc,g}$ and ${\mathbf{A}}_{g,g}$ are the partitions of the pedigree-relationship matrix $\mathbf{A}$ relating non-genotyped ancestors of genotyped animals and genotyped animals, and among genotyped animals, respectively. Finally, using ${\mathbf{J}}_{g}$ and ${\mathbf{J}}_{anc}$, ungenotyped animals that are not ancestors of genotyped animals receive a covariate value corresponding to the average of their parents’ covariate. After computing the covariates for all animals, the $\mathbf{J}$ covariates were fitted in the model as follows. The $\mathbf{J}$ covariate for the additive genetic effect corresponded to that of the animal itself, while the $\mathbf{J}$ covariate for the maternal genetic effect corresponded to that of its dam. Following Fernando et al. [49] and Hsu et al. [46], the product of an animal’s $\mathbf{J}$ covariate and the estimated regression coefficient was added to its estimated genetic value to compute the animal’s genomic EBV.

### Genetic and genomic connections among countries

In international evaluations, genetic connections among countries are mainly provided by common bulls (CB), i.e. sires that have recorded offspring in two or more countries. Therefore, we quantified the number of CB and common maternal grand-sires (CMGS, i.e. maternal grand-sires with recorded grand-offspring in two or more countries). Furthermore, we quantified the number of sires and dams with recorded offspring in each national pedigree. Then, for all these groups of animals, we also quantified whether a genotype was provided by the same country or provided by other countries. This shows the potential increases in genetic connectedness among countries over national evaluations and pedigree-based international evaluation due to the inclusion of genotypes provided by other countries in a ssSNPBLUP_*INT*_ evaluation.

### Validation

We used the linear regression (LR) method [50] to evaluate level and dispersion bias, as well as the population accuracy of the EBV to investigate the benefits of using genomic and international data over current international pedigree-based evaluations and national evaluations. Hereafter, we describe the LR method and its estimators that we used, and how these were applied in the above scenarios.

#### Linear regression method and estimators

The LR method [50] compares EBV for a group of individuals (called “focal group”) obtained in two evaluations: a partial evaluation (hereafter denoted by subscript $p$) and a whole evaluation (hereafter denoted by subscript $w$). In the partial evaluation, EBV (${\widehat{\mathbf{u}}}_{p}$) are estimated using less information, while in the whole evaluation, EBV (${\widehat{\mathbf{u}}}_{w}$) are estimated using more information. The following estimators from the LR method were calculated:

- Level bias (${\widehat{\Delta }}_{p}$): defined as the difference between the mean EBV under the evaluation $p$ and $w$ (${\widehat{\Delta }}_{p}={\overline{\widehat{\mathbf{u}}} }_{p}-{\overline{\widehat{\mathbf{u}}} }_{w}$). The expectation of ${\widehat{\Delta }}_{p}$ is 0 in absence of level bias. Level bias was expressed in national genetic standard deviations (${\widehat{\sigma }}_{u}$) for easier interpretation, i.e. as $\widehat{{\Delta }_{p}}/{\widehat{\sigma }}_{u}$.
- Dispersion bias (${\widehat{b}}_{p}$): defined as the slope of the regression of ${\widehat{\mathbf{u}}}_{w}$ on ${\widehat{\mathbf{u}}}_{p}$ and computed as ${\widehat{b}}_{p}= \frac{cov({\widehat{\mathbf{u}}}_{w}, {\widehat{\mathbf{u}}}_{p})}{{var(\widehat{\mathbf{u}}}_{p})}$. The expectation of ${\widehat{b}}_{p}$ is 1 in the absence of dispersion bias, while deviations from 1 indicate dispersion bias. We assumed that considerable under-dispersion was present when ${\widehat{b}}_{p}$ > 1.15 while considerable over-dispersion was present when ${\widehat{b}}_{p}$ < 0.85. We assumed that values within 15% from the optimal value of 1 were acceptable, similarly to Tsuruta et al. [51] and the Interbull genomic validation test [52].
- Accuracy of partial EBV (${\widehat{acc}}_{p}$): based on the covariance of ${\widehat{\mathbf{u}}}_{w}$ and ${\widehat{\mathbf{u}}}_{p}$ and computed as ${\widehat{acc}}_{p}= \sqrt{\frac{cov({\widehat{\mathbf{u}}}_{w}, {\widehat{\mathbf{u}}}_{p})}{\left(1-\overline{F }\right) {\widehat{\sigma }}_{u}^{2}}}$, where $\overline{F }$ is the mean inbreeding coefficient of the focal group computed from the international pedigree. ${\widehat{acc}}_{p}$ is an estimator of the accuracy of the partial EBV (${\widehat{\mathbf{u}}}_{p}$).
- Increases in population accuracies ($inc\_ac{c}_{p,w}$): the ratio of accuracy ${\widehat{\rho }}_{p,w}$ is defined as the Pearson correlation between ${\widehat{\mathbf{u}}}_{p}$ and ${\widehat{\mathbf{u}}}_{w}$ and computed as ${\widehat{\rho }}_{p,w}=$ $\frac{cov({\widehat{\mathbf{u}}}_{w}, {\widehat{\mathbf{u}}}_{p})}{\sqrt{{var(\widehat{\mathbf{u}}}_{p}){var(\widehat{\mathbf{u}}}_{w})}}$. ${\widehat{\rho }}_{p,w}$ has expectation equal to the ratio of the accuracies in the two evaluations ($\frac{{acc}_{p}}{{acc}_{w}}$), where $acc$ is defined as the correlation between the true breeding values (TBV) and the EBV across individuals in a population [50]. Consequently, ${1/\widehat{\rho }}_{p,w}$ represents the increase in accuracy obtained with evaluation $w$ [53]. We expressed the increase in accuracy relative to evaluation $p$ in percentage, i.e. ${inc\_ac{c}_{p,w}= (1/\widehat{\rho }}_{p,w}-1)\cdot 100\%$. For example, if ${\widehat{\rho }}_{p,w}$ is 0.80, the relative increase in population accuracy when moving from evaluation $p$ to $w$ is 25%.

The LR estimates were obtained using R statistical software [54] and their standard errors (SE) were obtained using bootstrapping (R “boot” package [55]) of individuals within each focal group. In total, 10,000 bootstrap samples were generated, where each sample was obtained by randomly drawing with replacement $N$ animals from the focal group, with $N$ being the number of animals in the focal group.

#### Application of the LR method

##### Focal group

The LR method can be applied to any focal group, defined as a homogenous group of individuals in a population, i.e. with prediction at the time of selection based on the same sources and amount of information [56]. For each country, we defined the focal group as the group of animals with both phenotypes and genotypes (irrespectively of which country provided the genotype) born from 2014 onwards. Animals born from 2014 onwards are assumed to be part of the last generation of the pedigree (up to 2019), with the generation interval estimated to be $\frac{{L}_{m}+{L}_{f}}{2}$ = 4.6 years, where ${L}_{m}$ and ${L}_{f}$ is the average generation interval for males and females, respectively. Table 4 reports the number of animals in each focal group for each country.

**Fig. 1:**
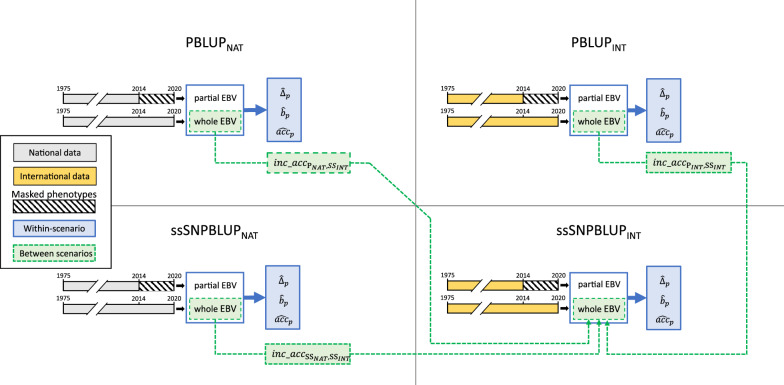
Schematic overview of the validation. Scenarios: *PBLUP*_*NAT*_ pedigree BLUP national, *ssSNPBLUP*_*NAT*_ single-step SNP-BLUP national, *PBLUP*_*INT*_ pedigree BLUP international, *ssSNPBLUP*_*INT*_ single-step SNP-BLUP international. For each scenario, a partial and a whole evaluation was carried out (timelines). National scenarios used only national data (grey timelines), while international scenarios used data from all countries (yellow timelines). In the partial evaluation, partial estimated breeding values (EBV) were obtained by masking the phenotypes of animals born from 2014 onwards (striped timeline). In the whole evaluation, whole EBV were obtained using all phenotypes. Within-scenario estimators of level bias (${\widehat{\Delta }}_{p}$), dispersion bias (${\widehat{b}}_{p}$), and accuracy of partial EBV (${\widehat{acc}}_{p}$) were obtained from the partial and whole EBV of each scenario (blue solid lines and boxes). Between scenarios increases in population accuracies ($inc\_acc$) of moving towards ssSNPBLUP_*INT*_ scenario were computed using the whole EBV of each scenario (green dotted lines and boxes)

Figure 1 shows a schematic overview of the application of the LR method within-scenario and between scenarios.

##### Within-scenario level and dispersion bias, and accuracy of partial EBV

LR estimators of level bias, dispersion bias, and accuracy of partial EBV were computed within-scenario for both the international and national scenarios. These estimators were computed by carrying out a whole and a partial evaluation for each scenario (Fig. 1). In the whole evaluation of each scenario all available data was used, whereas in the partial evaluation phenotypes of animals born from 2014 onwards were set to missing (Fig. 1). Pedigree and genomic data remained the same in both whole and partial evaluations. Thus, in both whole and partial evaluations, the same observed allele frequencies were used in the single-step models. Table 4 presents the number of phenotypes in the whole and partial evaluations of each scenario. Knowing the expected unbiased values described above, level and dispersion bias can be compared in each scenario to evaluate whether any changes in bias were introduced when moving from the current PBLUP_*INT*_ scenario to ssSNPBLUP_*INT*_ scenario and, in a similar way, whether the observed level and dispersion bias of international scenarios were already present in the national scenarios. Finally, the ${\widehat{acc}}_{p}$ provides an estimate of the changes of the accuracy of partial EBV in each scenario given the different sources of information used.

##### Increases in population accuracies between scenarios

To evaluate the benefits of using genomic information at the international level, we compared the increases in population accuracies ($inc\_ac{c}_{p,w}$) obtained when moving from either PBLUP_*NAT*_, ssSNPBLUP_*NAT*_, or PBLUP_INT_ scenarios to the ssSNPBLUP_*INT*_ scenario (Fig. 1). Following Legarra and Reverter [50], adding genotypes to pedigree-based models can be considered as additional information. Similarly, national scenarios can be viewed as evaluations with partial information, and international scenarios as evaluations with additional information which is represented by phenotypes and genotypes of relatives recorded in another country. The $inc\_ac{c}_{p,w}$ when moving towards ssSNPBLUP_*INT*_ were computed using the EBV from the whole evaluations (${\widehat{\mathbf{u}}}_{w}$) of each scenario (Fig. 1). Thus, ${inc\_acc}_{{\mathrm{P}}_{NAT}, {\mathrm{SS}}_{INT}}$ estimates the increase in population accuracy from PBLUP_*NAT*_ towards ssSNPBLUP_*INT*_; $inc\_ac{c}_{{\mathrm{SS}}_{NAT}, {\mathrm{SS}}_{INT}}$ estimates the increase from ssSNPBLUP_*NAT*_ to ssSNPBLUP_*INT*_; and ${inc\_acc}_{{\mathrm{P}}_{INT}, {\mathrm{SS}}_{INT}}$ estimates the increase from PBLUP_*INT*_ to ssSNPBLUP_*INT*_. Differences in observed allele frequencies that could be present between ssSNPBLUP_*NAT*_ and ssSNPBLUP_*INT*_ whole evaluations are accounted for by the two $\mathbf{J}$ covariates [46] (for additive and maternal genetic effects).

### Software and settings

EBV were computed using the MiXBLUP software [57] instruction files for PBLUP_*INT*_ and ssSNPBLUP_*INT*_ are reported in Additional file 2: Files S2 and S3, respectively. The convergence criterion for the preconditioned conjugate gradient (PCG) algorithm for iteratively solving the mixed model equations was defined as the square root of the relative difference between solutions of two consecutive PCG iterations and was set to 10^–5^. To ensure that all EBV were expressed against the same base, EBV were scaled relative to a base generation common to all scenarios, which was defined in each country as the group of national animals born in 2002 with an available AWW phenotype. All validation results were computed using these scaled EBV.

## Results

Hereafter, we first present the results on genetic and genomic connections among countries, followed by the LR estimates computed within-scenario for ssSNPBLUP_*INT*_, and the differences with those estimates computed for the other scenarios implemented. Finally, we present the results on the increases in population accuracies between scenarios.

### Genetic and genomic connections among countries

**Fig. 2:**
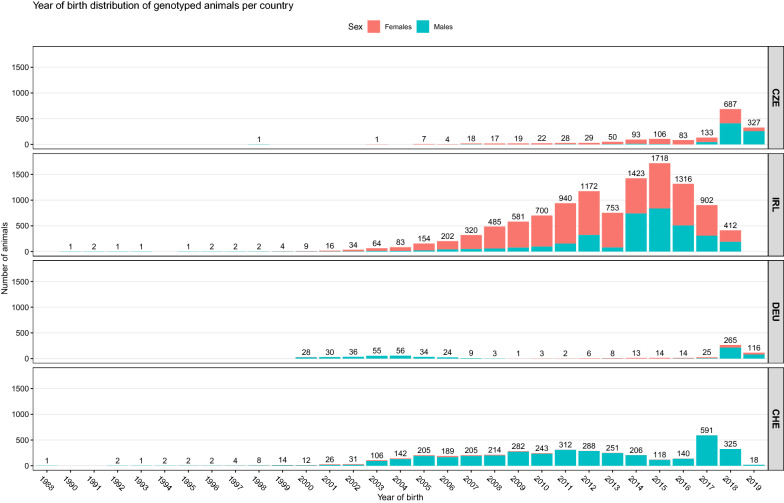
Number of genotyped animals (on the y-axis) per year of birth (on the x-axis) and sex (red = females, blue = males) in each country. *CZE* Czech Republic, *IRL* Ireland, *DEU* Germany, *CHE* Switzerland

**Fig. 3:**
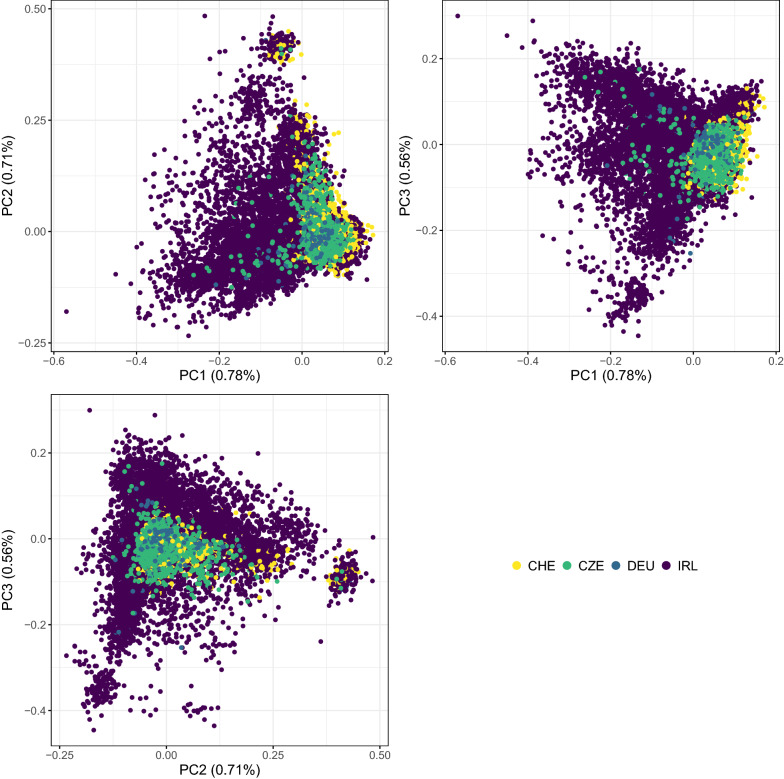
Plot of the first three principal components (PC) and percentage of explained variance (within brackets) of the genomic relationship matrix. Colours indicate the country sending the genotype. *CHE* Switzerland, *CZE* Czech Republic, *DEU* Germany, *IRL* Ireland

The distribution of genotyped animals varied between countries. Most of the genotyped animals were born after 2000, with an overall increasing genotyping trend during more recent years (Fig. 2). In particular, in CZE and DEU, 88 and 60% of the genotyped animals were born from 2014 onwards. Overall, genotyped animals were 51.5% males and 48.5% females. The sex ratio of the genotyped animals differed between countries, with 45%, 31%, 77%, and 95% of the genotypes being males in CZE, IRL, DEU, and CHE, respectively. Finally, PCA shows that the populations were genetically close and no specific population clusters were observed (Fig. 3).

The number of genotyped sires ranged from 57 for DFS to 1166 for IRL, and the number of genotyped dams from 68 for DEU to 4190 for IRL with DFS having no genotyped dams (Table 5). In IRL, DEU and CHE the majority of genotyped sires in the national pedigree were genotyped by the country itself. Nonetheless, the number of sires with a genotype provided by another country ranged from 24 of IRL to 110 of CZE (equal to 83% of the total genotyped sires in CZE). Interestingly, DFS, which did not provide genotypes, was associated with 57 genotyped sires, thanks to genotypes provided by other countries. The proportion of genotyped sires that had recorded offspring was 57% for CZE, 79% for DFS and > 90% for IRL, DEU and CHE. Except for IRL, genotyped sires had a larger average number of recorded offspring compared to that of all sires with records. The number of genotyped sires with more than 100 recorded offspring in at least five herds (which provides an indication of the sires that may be used in artificial insemination) was small in all countries (< 15 sires). Finally, almost all genotyped dams in the national pedigree were genotyped by the country itself and only a small number was genotyped by another country (2, 8 and 3 for CZE, DEU and CHE, respectively).

**Table 5:** Overview of recorded offspring per sires and dams, and number of genotyped sires and dams in each national pedigree

	CZE	DFS	IRL	DEU	CHE
Sires					
With recorded offspring	720	4591	9341	5283	1892
Average recorded offspring	19.3	21.1	7.3	22.2	18.9
≥ 20 recorded offspring	220	1399	600	1855	518
≥ 100 recorded offspring	15	162	59	163	63
≥ 100 recorded offspring (in at least 5 herds)	11	157	57	43	38
Sires with genotype					
Number	132	57	1166	368	956
Genotyped by the country itself	22	–	1142	273	863
Genotyped by another country	110	57	24	95	93
With recorded offspring	75	45	1100	350	856
Average recorded offspring	28.1	30.7	6.3	48.0	22.1
≥ 20 recorded offspring	34	23	51	273	295
≥ 100 recorded offspring	4	4	7	35	32
≥ 100 recorded offspring (in at least 5 herds)	4	4	7	10	14
Dams					
With recorded offspring	4457	30,212	47,334	35,340	9785
Average recorded offspring	3.1	3.2	1.4	3.3	3.6
Dams with genotype					
Number	375	–	4190	68	185
With recorded offspring	355	–	3311	58	181
Average recorded offspring	2.9	–	1.4	2.9	6.5

*CZE* Czech Republic; *DFS* Denmark Finland and Sweden; *IRL* Ireland; *DEU* Germany; *CHE* Switzerland

We quantified the total number of CB and CMGS that had recorded offspring in two or more countries. In total, there were 422 CB, of which 106 were genotyped, and 642 CMGS of which 72 were genotyped. The average number of CB between countries was 95, ranging from 38 for CZE-CHE to 155 for DFS-DEU (Table 6). On average across pairs of countries, 33 CB were also genotyped, ranging from 20 for CZE-CHE to 49 for IRL-DEU. The average number of CMGS between countries was 124 ranging from 58 for DFS-CHE and IRL-CHE to 235 for DEU-CHE (Table 6). On average across pairs of countries, 19 CMGS were genotyped, ranging from 11 for CZE-CHE to 36 for DEU-CHE.

**Table 6:** Number of (genotyped) common bulls (CB) and (genotyped) common maternal grand-sires (CMGS) connecting each pair of countries

Pair of countries		CB		CMGS	
		Number	With genotype	Number	With genotype
CZE	DFS	77	24	92	17
CZE	IRL	87	43	82	16
CZE	DEU	133	38	189	30
CZE	CHE	38	20	72	11
DFS	IRL	102	32	114	15
DFS	DEU	155	37	190	21
DFS	CHE	40	21	58	13
IRL	DEU	142	49	149	22
IRL	CHE	41	22	58	12
DEU	CHE	131	47	235	36

Details on countries sending the genotype for CB or CMGS are in Additional file 1: Table S7

*CZE* Czech Republic; *DFS* Denmark, Finland and Sweden; *IRL* Ireland; *DEU* Germany; *CHE* Switzerland

### Within-scenario bias and accuracy of EBV

Bias and accuracy of EBV were calculated comparing partial and whole EBV of each scenario. Overall, ssSNPBLUP_*INT*_ showed negative level bias (${\widehat{\Delta }}_{p}$) for direct EBV and small ${\widehat{\Delta }}_{p}$ for maternal EBV (Table 7). For direct EBV, the average ${\widehat{\Delta }}_{p}$ across countries was − 0.17 genetic standard deviations (GSD), ranging from − 0.22 GSD for CZE to − 0.10 GSD for IRL. For maternal EBV, the average ${\widehat{\Delta }}_{p}$ across countries was 0.02 GSD, ranging from − 0.02 GSD for DEU to 0.06 GSD for CHE. Overall, direct EBV were considerably over-dispersed in all countries except for IRL: ${\widehat{b}}_{p}$ was on average 0.83 across countries, ranging from 0.79 for CZE to 0.87 for IRL. For maternal EBV, ${\widehat{b}}_{p}$ was on average 0.88 across countries and showed considerable over-dispersion only for DEU, while other countries showed over-dispersion but remained within the 0.85–1.15 interval. The average ${\widehat{acc}}_{p}$ across countries for ssSNPBLUP_*INT*_ was 0.36 (ranging from 0.35 for CZE, IRL and DEU to 0.40 for CHE) and 0.25 (ranging from 0.23 for CZE and DEU to 0.29 for CHE), for direct and maternal EBV, respectively.

**Table 7:** Level bias (${\widehat{\Delta }}_{p}$), dispersion bias (${\widehat{b}}_{p}$) and accuracy of partial EBV (${\widehat{acc}}_{p}$) of direct and maternal EBV for the focal group, in each scenario and for each country

Country	Direct				Maternal			
	PBLUP_*NAT*_	ssSNPBLUP_*NAT*_	PBLUP_*INT*_	ssSNPBLUP_*INT*_	PBLUP_*NAT*_	ssSNPBLUP_*NAT*_	PBLUP_*INT*_	ssSNPBLUP_*INT*_
$${\widehat{\Delta }}_{p}$$(GSD)								
CZE	− 0.25	− 0.23	− 0.23	− 0.22	− 0.01	− 0.03	0.00	0.01
IRL	− 0.08	− 0.10	− 0.10	− 0.10	− 0.02	0.01	− 0.01	0.02
DEU	− 0.19	− 0.10	− 0.21	− 0.15	− 0.06	− 0.02	− 0.05	− 0.02
CHE	− 0.28	− 0.27	− 0.23	− 0.21	0.04	0.03	0.04	0.06
Range of SE	0.01–0.02	0.01–0.02	0.01–0.02	0.01–0.02	0.00–0.01	0.00–0.01	0.00–0.01	0.00–0.01
$${\widehat{b}}_{p}$$								
CZE	0.72	0.76	0.65	0.79	1.04	0.94	1.06	0.96
IRL	0.96	0.87	1.00	0.87	0.92	0.87	0.91	0.85
DEU	0.79	0.85	0.77	0.82	0.78	0.79	0.79	0.79
CHE	0.80	0.79	0.80	0.82	1.06	0.98	0.99	0.93
Range of SE	0.02–0.07	0.02–0.06	0.02–0.06	0.02–0.04	0.02–0.07	0.02–0.07	0.02–0.07	0.02–0.05
$${\widehat{acc}}_{p}$$								
CZE	0.23	0.25	0.25	0.35	0.17	0.17	0.19	0.23
IRL	0.23	0.29	0.26	0.35	0.17	0.22	0.18	0.24
DEU	0.26	0.31	0.27	0.35	0.18	0.20	0.18	0.23
CHE	0.34	0.38	0.35	0.40	0.22	0.27	0.24	0.29
Range of SE	0.00–0.02	0.00–0.02	0.00–0.02	0.01–0.02	0.00–0.01	0.00–0.01	0.00–0.01	0.00–0.01

Level bias is expressed in genetic standard deviations (GSD)

Focal group: animals with phenotypes and genotypes born from 2014 onwards

Range of SE: minimum and maximum Standard Error across countries in each scenario (all standard errors are reported in Additional file 1: Table S8)

*PBLUP*_*NAT*_ pedigree-based BLUP national, *ssSNPBLUP*_*NAT*_ single-step SNP-BLUP national, *PBLUP*_*INT*_ pedigree-based BLUP international, *ssSNPBLUP*_*INT*_ single-step SNP-BLUP international

*CZE* Czech Republic; *DFS* Denmark, Finland and Sweden; *IRL* Ireland; *DEU* Germany; *CHE* Switzerland

Overall, ssSNPBLUP_*INT*_ performed better than PBLUP_*NAT*_ based on level bias, dispersion bias, and accuracy. Indeed, for direct EBV, ssSNPBLUP_*INT*_ showed less level bias and less over-dispersion (albeit not statistically significant) compared to PBLUP_*NAT*_, with values of ${\widehat{\Delta }}_{p}$ improving by 0.03 GSD on average across countries, and ${\widehat{b}}_{p}$ being closer to 1 in all countries (except for IRL) (Table 7). For maternal EBV, ssSNPBLUP_*INT*_ showed similar level bias compared to PBLUP_*NAT*_: difference in ${\widehat{\Delta }}_{p}$ of 0.00 GSD on average across countries. However, maternal EBV were more over-dispersed in ssSNPBLUP_*INT*_ compared to PBLUP_*NAT*_: average difference in ${\widehat{b}}_{p}$ of − 0.07. In CZE and CHE, ${\widehat{b}}_{p}$ went from small under-dispersion of PBLUP_*NAT*_ to small over-dispersion of ssSNPBLUP_*INT*_. Finally, in all countries the accuracy of partial EBV was greater in ssSNPBLUP_*INT*_ than in PBLUP_*NAT*_: on average across countries, the difference in ${\widehat{acc}}_{p}$ between scenarios was 0.10 and 0.06 for direct and maternal EBV, respectively.

Overall, on average across countries, ssSNPBLUP_*INT*_ showed similar level bias and dispersion bias for both direct and maternal EBV compared to ssSNPBLUP_*NAT*_, but ssSNPBLUP_*INT*_ did have a higher accuracy (Table 7). In all countries, the accuracy of partial EBV was greater with ssSNPBLUP_*INT*_ compared to ssSNPBLUP_*NAT*_: on average across countries, the difference in ${\widehat{acc}}_{p}$ between scenarios was 0.06 and 0.03 for direct and maternal EBV, respectively.

Overall, on average across countries, ssSNPBLUP_*INT*_ resulted in similar or less level bias, similar dispersion bias, and higher accuracy than PBLUP_*INT*_. Indeed, for direct EBV, ssSNPBLUP_*INT*_ showed similar or less level bias compared to PBLUP_*INT*_: ${\widehat{\Delta }}_{p}$ improved by 0.02 GSD on average across countries, with the largest improvement observed for DEU (0.06 GSD) (Table 7). For maternal EBV, ssSNPBLUP_*INT*_ showed a similar level bias as PBLUP_INT_. In all countries except for IRL, direct EBV showed less over-dispersion in ssSNPBLUP_*INT*_ compared to PBLUP_*INT*_ with values of ${\widehat{b}}_{p}$ being closer to 1. In IRL more over-dispersion of direct EBV was observed in ssSNPBLUP_*INT*_ compared to PBLUP_*INT*_ although ${\widehat{b}}_{p}$ remained within the 0.85–1.15 interval. Maternal EBV showed similar or more over-dispersion in ssSNPBLUP_*INT*_ compared to PBLUP_*INT*_. In CZE, ${\widehat{b}}_{p}$ for maternal EBV went from small under-dispersion of PBLUP_*INT*_ to small over-dispersion of ssSNPBLUP_*INT*_. Finally, in all countries, the accuracy of partial EBV was higher with ssSNPBLUP_*INT*_ compared to PBLUP_*INT*_: on average across countries, the difference in ${\widehat{acc}}_{p}$ between scenarios was 0.08 and 0.05 for direct and maternal EBV, respectively.

### Increases in population accuracies between scenarios

Increases in population accuracies ($inc\_acc$) were observed in all countries when moving from any scenario to the ssSNPBLUP_*INT*_ scenario (Table 8). When moving from PBLUP_*NAT*_ to ssSNPBLUP_*INT*_, ${inc\_acc}_{{P}_{NAT},{\mathrm{SS}}_{INT}}$ was 14.9% (ranging from 9.2% for CZE to 27.2% for IRL) and 33.0% (ranging from 19.0% for DEU to 47.8% for IRL) on average across countries for direct and maternal EBV, respectively. When moving from ssSNPBLUP_*NAT*_ to ssSNPBLUP_*INT*_, ${inc\_acc}_{{SS}_{NAT},{\mathrm{SS}}_{INT}}$ was 6.2% (ranging from 3.4% for CHE to 9.3% for DEU) and 16.8% (ranging from 12.4% for DEU to 25.6% for CZE) on average across countries for direct and maternal EBV, respectively. Finally, when moving from the current PBLUP_*INT*_ to ssSNPBLUP_*INT*_, ${inc\_acc}_{{P}_{INT},{\mathrm{SS}}_{INT}}$ was 13.7% (ranging from 8.5% for CZE to 25.0% for IRL) and 25.8% (ranging from 16.5% for DEU to 41.8% for IRL) on average across countries for direct and maternal EBV, respectively.

**Table 8:** Increases in population accuracy ($inc\_acc$)^a^ of moving from each scenario to ssSNPBLUP_*INT*_, for direct and maternal estimated breeding values (EBV) in the focal group for each country

Country	Direct			Maternal		
	PBLUP_*NAT*_	ssSNPBLUP_*NAT*_	PBLUP_*INT*_	PBLUP_*NAT*_	ssSNPBLUP_*NAT*_	PBLUP_*INT*_
CZE	9.2	5.6	8.5	32.8	25.6	19.5
IRL	27.2	6.8	25.0	47.8	14.1	41.8
DEU	13.1	9.3	11.4	19.0	12.4	16.5
CHE	10.3	3.4	9.8	32.3	14.9	25.3
Range of SE	0.6–1.5	0.2–1.0	0.6–1.3	2.0–2.8	0.6–2.1	1.5–2.1

Focal group: animals with phenotypes and genotypes born from 2014 onwards

Range of SE: minimum and maximum standard error across countries in each scenario

*PBLUP*_*NAT*_ pedigree-based BLUP national, *ssSNPBLUP*_*NAT*_ single-step SNP-BLUP national, *PBLUP*_*INT*_ pedigree-based BLUP international, *ssSNPBLUP*_*INT*_ single-step SNP-BLUP international

*CZE* Czech Republic; *DFS* Denmark, Finland and Sweden; *IRL* Ireland; *DEU* Germany; *CHE* Switzerland

^a^Increases in population accuracies are expressed in % relative to each scenario whole EBV

## Discussion

In this study, we developed an international single-step SNPBLUP genomic evaluation for beef cattle and investigated the benefits of including genomic data in the current pedigree-based international evaluations. Hereafter, we first discuss the possible benefits of single-step evaluations to increase the existing pedigree genetic connectedness among countries. Then, we discuss the increases in accuracies of EBV due to the inclusion of genomic data in international evaluations, followed by its impact on level and dispersion bias compared to both current pedigree-based international evaluations and national evaluations. Finally, we discuss the possible implications of this study for beef cattle international evaluations.

### Connectedness among countries

In international evaluations, straightforward measures are used to quantify genetic connectedness between countries, such as the number of CB and CMGS [30, 58, 59]. Table 6 shows that an average of 39 and 17% of CB and CMGS among country pairs were also genotyped. In addition, Table 5 shows an increase of genotyped sires within each country when combining genomic data in international genomic evaluations, especially for DFS and CZE that have no or a small number of genotyped sires at the national level. Genomic data can help to reveal existing relationships between animals that would otherwise appear as disconnected according to pedigree data, but also by refining relationships that are observed in the pedigree based on the captured Mendelian sampling [10, 27]. Additional file 3: Fig. S1 shows that genomic data help to differentiate existing pedigree relationships among genotyped animals, which are also extended to ungenotyped animals in single-step approaches [29]. Sophisticated measures of genetic connectedness require the direct inverse of the left-hand side of mixed models equations or its approximation [60, 61], making them computationally very demanding and possibly not applicable to large datasets. Nonetheless, it is expected that genotyping CB and CMGS and using genomic data in international evaluations would increase connectedness between countries. For instance, Yu et al. [27] showed that genomic data increase connectedness between management units (e.g. herds) compared to pedigree data.

### Benefits of international single-step genomic evaluations

We used the LR method to validate results within and between scenarios as it presents several advantages compared to other validation methods. The LR method can be applied to multi-trait models and traits where the animals’ phenotype is not available for all environments [50, 53, 62]. One of the main advantages of the LR method is that it does not require pre-correction of phenotypes, which are particularly difficult to define for maternally-affected traits [23], allowing for validation of both direct and maternal effects [50, 53].

Our results showed that ssSNPBLUP_*INT*_ improved accuracies relative to PBLUP_*INT*_, which is in line with the results of VanRaden and Sullivan [63] and Jorjani et al. [20], and also relative to ssSNPBLUP_*NAT*_, which is in line with the results of Lund et al. [19]. Even for countries with the largest number of genotyped animals with phenotypes at the national level, such as IRL and CHE in this study, we observed increases in population accuracies (Table 8). Increases in population accuracies for IRL and CHE may be related to two factors: (1) a larger multi-country reference population compared to national ones (e.g. in international single-step evaluations the number of genotyped animals with phenotypes for IRL increased by 102% relative to national evaluations; Table 1), and (2) the inclusion via single-step international evaluations of phenotypic information on relatives recorded in other countries and connected via sires, CB and CMGS (Tables 5 and 6). The observed benefits of sharing genotypes across countries were also confirmed by the accuracy of partial EBV (which, unlike $inc\_acc$, does not consider the phenotypes of animals in the focal group) with values of ${\widehat{acc}}_{p}$ being the highest under ssSNPBLUP_*INT*_: on average across countries 0.36 and 0.25, for direct and maternal EBV, respectively (Table 7). Overall, ${\widehat{acc}}_{p}$ of ssSNPBLUP_*INT*_ were closer to those of ssSNPBLUP_*NAT*_ than to those of PBLUP_*INT*_, showing that genomic information should be considered in international evaluations.

Genomic information is expected to increase the accuracy for genotyped animals due to increases in the variation in relationships between animals and by better capturing variation in Mendelian sampling. Using a single-step approach, the benefits of genomic information are also propagated to ungenotyped animals [29]. This was reflected in higher ${\widehat{acc}}_{p}$ under ssSNPBLUP_*INT*_ compared to other scenarios for animals with phenotypes but no genotypes born from 2014 onwards, and in increases in population accuracies (albeit small) for these animals when moving from any other scenarios to ssSNPBLUP_*INT*_ (see Additional file 1: Tables S5 and S6). The increases in accuracies for DFS, which did not provide genotypes, show the potential benefits of international single-step evaluations for countries with no genomic data available yet at the national level.

To our knowledge, this is the first published study that investigates bias in Interbeef evaluations. We evaluated whether moving towards genomic international models may introduce any level and dispersion bias compared to either international pedigree-based evaluation or national evaluations. Overall, ssSNPBLUP_*INT*_ had a similar level and dispersion bias compared to either PBLUP_*INT*_ or national scenarios. Across countries and scenarios, direct EBV showed negative level bias and considerable over-dispersion (${\widehat{b}}_{p}$ < 0.85) (except for IRL), while maternal EBV showed level bias close to 0 GSD and dispersion within the 0.85–1.15 interval (except for DEU). As expected, the largest SE of the LR estimates were observed for DEU that has the smallest number of animals in the focal group among countries (see Additional file 1: Table S8). Across scenarios, IRL showed the lowest level and dispersion bias compared to other countries; this result could be related to IRL having the largest number of genotypes among countries. These results also underline the importance of formal validation procedures for current Interbeef international evaluations. We further investigated possible differences in genetic level between ssSNPBLUP_*INT*_ and PBLUP_*INT*_ using genetic trends of sires with at least 10 recorded offspring in a country and including at least five sires per year, similarly to Venot et al. [64] (see Additional file 3: Fig. S2). If selective genotyping is present, genetic trends between ssSNPBLUP_*INT*_ and PBLUP_*INT*_ can differ [65]. Overall, genetic trends overlapped between ssSNPBLUP_*INT*_ and PBLUP_*INT*_ for all countries except for a systematic difference in DEU for direct effects, and in CHE for both direct and maternal effects. CHE had almost 55% of the sires with at least 10 recorded offspring genotyped, while in other countries this number ranged from about 1% (DFS) to 15% (CZE). Differences for DEU and CHE could be related to selective genotyping. Differences in genetic trends between pedigree-based and single-step evaluations were already present for national scenarios and reduced for international scenarios (results not shown). Overall, the results of this study suggest that level and dispersion bias of national evaluations will remain similar or slightly decrease with international single-step genomic evaluations.

So far, only few national studies reported LR estimates in beef cattle, in particular for weaning weight. Overall, we observed improvements in ${\widehat{acc}}_{p}$ when including genomic information in national scenarios. On average across countries, the ${\widehat{acc}}_{p}$ of PBLUP_*NAT*_ scenarios was 0.26 (ranging from 0.23 for CZE to 0.34 for CHE) for direct EBV and 0.19 for maternal EBV (ranging from 0.17 for CZE to 0.22 for CHE). In ssSNPBLUP_*NAT*_, ${\widehat{acc}}_{p}$ was on average 0.31 for direct EBV (ranging from 0.25 for CZE to 0.38 for CHE) and 0.21 for maternal EBV (ranging from 0.17 for CZE to 0.27 for CHE) (Table 7). In Brazilian Angus, Campos et al. [66] conducted pedigree and genomic evaluations for growth traits using ssGBLUP [7, 8] with about 1600 genotyped animals. For weaning weight gain, the average ${\widehat{acc}}_{p}$ across validation groups was 0.39 for direct effect and 0.30 for total maternal (weaning weight and tick count) for PBLUP, and 0.45 for direct effect and 0.37 for total maternal for ssGBLUP. These values are in agreement with our results for countries such as CHE and smaller for other countries such as CZE. These differences could be due to the use of a multi-variate model in combination with other growth traits (birth weight and post-weaning weight) in Campos et al. [66] as well as differences in population structure and trait definition. Recently, Jang et al. [67] reported LR estimates for genomic predictions of weaning weight in American Angus using a large reference population of about 180,000 genotyped animals and over 2,4 million weaning weight phenotypes. Using ssGBLUP with similar modelling as that used here (“M1” in their study), they found high values of ${\widehat{acc}}_{p}$ of 0.72 for direct EBV and 0.62 for maternal EBV. These results confirm that the use of large reference populations enables to achieve high weaning weight accuracies for both direct and maternal EBV in young animals. In contrast with the results of our study, both Jang et al. [67] and Campos et al. [66] reported values of dispersion for weaning weight that were mostly within the 0.85–1.15 interval, except for pedigree evaluations of total maternal in Campos et al. [66].

The negative level bias for direct EBV and its associated over-dispersion may be related to selective genotyping for animals composing the focal group [46, 47]. Negative values of ${\widehat{\Delta }}_{p}$ indicate a higher mean EBV under whole evaluations compared to partial evaluations. This could be related to the low genotyping rate of animals born from 2014 onwards, i.e. 15%, 17%, 1% and 10% in CZE, IRL, DEU and CHE, respectively. Thus, genotyped animals used in the focal group could be a group of selected individuals with higher EBV for weaning weight compared to those born in the same generation and with no genotype. When the focal group was composed of animals born from 2014 onwards with phenotypes but no genotypes, level and dispersion bias were on average closer to the unbiased values of 0 and 1, respectively (see Additional file 1: Table S5). We further investigated the possible presence of selective genotyping using the countries’ realized Mendelian sampling (RMS) trends under both PBLUP_*INT*_ and ssSNPBLUP_*INT*_ scenarios (see Additional file 4: Fig. S3) for genotyped animals (with or without phenotypes) and ungenotyped animals (animals with phenotype in the country). Overall, the RMS trends of genotyped and ungenotyped animals of ssSNPBLUP_*INT*_ followed those of PBLUP_*INT*_ (see Additional file 4: Fig. S3). Following Abdollahi-Arpanahi et al. [65], the expectation of RMS is 0 when genotyped animals are a random sample of the population. Instead, RMS deviates from 0 with selective genotyping, i.e. when genotyped animals are selected based on information collected on the animal itself or its progeny. In this study, the RMS trends of all countries except for IRL showed that genotyped animals had non-zero and often positive RMS compared to ungenotyped animals, which generally had zero RMS (see Additional file 4: Fig. S3). This could be related to CZE, DEU and CHE starting to genotype animals more recently and potentially focussing on genotyping elite animals first. On the other hand, in IRL, genotyped animals overall showed almost no deviation in RMS trends, which suggests the absence of selective genotyping. This could be explained by IRL having a large number of genotyped animals and that the majority of them were females. Thus, results on the RMS trends seem to confirm the presence of selective genotyping in all countries except for IRL, and indeed suggest that the observed level and dispersion bias are due to selective genotyping.

### Implications

All countries in this study, except for IRL, do not have a national genomic evaluation in place for Limousin AWW and therefore scenario PBLUP_*NAT*_ represents their current national evaluations. In practice, deviations between national evaluations and pseudo-national scenarios are expected as pseudo-national scenarios used a subset of the international pedigree, which likely is more complete than the pedigree used in national evaluations, and because national evaluations are usually multi-trait. Another possible difference between pseudo-national scenarios and national evaluations is that the latter may use genetic groups to model missing pedigree information by fitting unknown parent groups (UPG, [68, 69]). Similarly to the current Interbeef pedigree-based international evaluations, both international and national scenarios in this study did not use genetic groups. Further research could investigate how UPG or metafounders [70] should optimally be defined to be used in (inter)national evaluations and whether or not fitting them in PBLUP or ssSNPBLUP helps to reduce the observed level and dispersion bias [71].

The increasing genotyping trend observed at the national level (Fig. 2) implies the need for the current Interbeef evaluation to consider also genomic data in the near future. In this study, we showed the feasibility of implementing a single-step evaluation at the international level using Limousin weaning weight data. The proposed international single-step evaluation approach is feasible also for other traits and breeds currently evaluated, i.e. Limousin, Charolais, Angus, Hereford and Simmental, provided that genotypes are available. The AWW is a representative trait for those that are currently evaluated traits in Interbeef, which are all maternally-affected traits, i.e. weight traits (composed by AWW [59]) and calving traits (composed by calving ease and birth weight [72]). Thus, we expect that similar benefits of implementing a single-step international evaluation could be observed for other breeds and traits, with larger benefits expected for traits with a low heritability [9, 16]. Moreover, we expect that increases in accuracies for ssSNPBLUP_*INT*_ could be further improved by including more genomic data, e.g. by increasing the number of participating countries. The ssSNPBLUP approach used in this study was shown to be applicable to large amounts of data while being computationally attractive [45, 73]. In this study, ssSNPBLUP_*INT*_ took 568 iterations and 23 min to converge using 10 CPUs Intel Xeon E5-1650v4 (3.60 GHz) and 4 GB of RAM, and an appropriate two-level PCG method [74].

The proposed international single-step approach requires sharing genotypes and phenotypes at the international level, which is subject to some limitations. For instance, in the Genomic MACE Service released by Interbull Centre for the Holstein breed, the international genomic EBV of young bulls are computed from national genomic EBV provided by participating countries [63] to avoid sharing raw data. To overcome such limitations and sensitivities around genotype data exchange, platforms have been developed to efficiently and safely share genotypes at the international level, e.g. GenoEX [75]. When sharing genotypes is not possible due to political or privacy limitations, an approximate single-step method could be used in which SNP effects and summary statistics are shared across countries and used jointly with raw pedigree and phenotype. Similar approaches have been proposed for international dairy cattle evaluations, e.g. [76–78].

## Conclusions

We developed an international single-step SNPBLUP genomic evaluation for beef cattle using Limousin weaning weight data and investigated the benefits of using genomic data compared to current pedigree-based evaluations. Combining multi-country genomic data in a single-step approach has the potential to increase existing pedigree-based genetic connectedness among countries via genotyped animals. Single-step international evaluations showed to increase accuracies of EBV compared to current pedigree-based international evaluations for both large and small countries as well as for countries with different amounts of genotypes at the national level. In this study, the increase in population accuracy when moving from current pedigree-based international evaluations to single-step genomic evaluation was on average across countries 13.7% and 25.8% for direct and maternal EBV, respectively. Moreover, increases in accuracies were observed for non-genotyped animals and countries without genotypes at the national level. Level and dispersion bias of international single-step genomic evaluations were similar or slightly reduced compared to current pedigree-based international and national (genomic) evaluations. The proposed international single-step approach can be applied to other traits and breeds allowing countries to improve the accuracies of their genetic evaluations.

## Supplementary Information

**Additional file 1: Table S1.** Number of phenotypes (N), minimum, mean, maximum, and phenotypic standard deviation (${\sigma }_{P}$) of males and females per country. **Table S2.** List of environmental effects in the national model of each country. **Table S3.** Direct and maternal genetic covariances (below diagonal), genetic variances (diagonal) and genetic correlations (above diagonal) within and across countries. **Table S4. **National genetic, environmental and residual (co)variances. **Table S5.** Level bias (${\widehat{\Delta }}_{p}$), dispersion bias (${\widehat{b}}_{p}$) and accuracy of partial EBV (${\widehat{acc}}_{p}$) of direct and maternal EBV for animals with phenotypes and no genotypes born from 2014 onwards, in each scenario and for each country. **Table S6.** Increases in population accuracy ($inc\_acc$) of moving from each scenario to ssSNPBLUP_*INT*_ for direct and maternal EBV for animals with phenotypes and no genotypes born from 2014 onwards for each country. **Table S7.** Countries sending the genotypes for Common Bulls (CB) and Common Maternal Grand-Sires (CMGS). **Table S8.** Level bias (${\widehat{\Delta }}_{p}$), dispersion bias (${\widehat{b}}_{p}$) and accuracy of partial EBV (${\widehat{acc}}_{p}$) of direct and maternal EBV for the focal group, in each scenario and for each country. Standard errors reported within parenthesis.

**Additional file 2: File S1.** Findhap instruction file. **File S2.** International pedigree-based BLUP MiXBLUP instruction file. **File S3.** International single-step SNPBLUP MiXBLUP instruction file.

**Additional file 3: Figure S1.** Plot of pedigree-based (x-axis) and genomic-based (y-axis) relationships between genotyped animals. The red dots indicate the relationships of the 41 genotypes removed due to pedigree incompatibilities. **Figure S2.** Differences in genetic trends between ssSNPBLUP_*INT*_ and PBLUP_*INT*_ per country for sires with at least 10 recorded offspring in the country (only birth years with at least 5 sires are included).

**Additional file 4: Figure S3.** Direct and maternal EBV realized Mendelian sampling (RMS) trends in each country^a^ expressed in genetic standard deviation (GSD) for genotyped and non-genotyped animals^b^ computed with pedigree-based BLUP international (PBLUP_*INT*_) and single-step SNP-BLUP international (ssSNPBLUP_*INT*_) models. ^a^*CZE* Czech Republic, *DFS* Denmark, Finland and Sweden, *IRL* Ireland, *DEU* Germany, *CHE* Switzerland. ^b^Genotyped animals: animals with genotype that appear in the pseudo-national pedigree (with or without phenotype in the country). Non-genotyped animals: animals that appear in the pseudo-national pedigree without genotype and with phenotype in the country.

## Data Availability

All information supporting the results are included in this article and its additional files. The data that support the findings of this study are available at Interbeef. Restrictions apply to the availability of these data, which were used under license for the current study.
