# Supplementary material for: International single-step SNPBLUP beef cattle evaluations for Limousin weaning weight
Source: Genet Sel Evol. 2022 Sep 4;54:57. doi: 10.1186/s12711-022-00748-0 (PMC9441073; doi:10.1186/s12711-022-00748-0)
Supplement: Supplementary file 1 — Additional file 1: Table S1. Number of phenotypes (N), minimum, mean, maximum, and phenotypic standard deviation (${\sigma }_{P}$σP) of males and females per country. Table S2. List of environmental effects in the national model of each country. Table S3. Direct and maternal genetic covariances (below diagonal), genetic variances (diagonal) and genetic correlations (above diagonal) within and across countries. Table S4. National genetic, environmental and residual (co)variances. Table S5. Level bias (${\widehat{\Delta }}_{p}$Δ^p), dispersion bias (${\widehat{b}}_{p}$b^p) and accuracy of partial EBV (${\widehat{acc}}_{p}$acc^p) of direct and maternal EBV for animals with phenotypes and no genotypes born from 2014 onwards, in each scenario and for each country. Table S6. Increases in population accuracy ( [file 12711_2022_748_MOESM1_ESM.docx]

**Additional file 1**

**Table S1 Number of phenotypes (N), minimum, mean, maximum, and phenotypic standard deviation (**$\boldsymbol{\sigma}_{\boldsymbol{P}}$**) of males and females per country ^a^.**

| COU ^a^ | Males | | | | | Females | | | | |  |
| --- | --- | --- | --- | --- | --- | --- | --- | --- | --- | --- | --- |
|  | N | Min | Mean | Max | $\sigma_{P}$ | N | Min | Mean | Max | $\sigma_{P}$ | |
| CZE | 6,816 | 173 | 293.2 | 411 | 37.8 | 7,076 | 157 | 262.8 | 366 | 33.5 | |
| DFS | 48,340 | 112 | 240.6 | 369 | 41.5 | 48,331 | 107 | 213.3 | 319 | 34.1 | |
| IRL | 40,873 | 134 | 297.2 | 460 | 53.9 | 27,213 | 127 | 264.4 | 402 | 45.2 | |
| DEU | 58,716 | 137 | 269.9 | 402 | 43.3 | 58,533 | 128 | 242.3 | 356 | 37.0 | |
| CHE | 18,197 | 112 | 233.4 | 354 | 39.2 | 17,498 | 107 | 209.7 | 312 | 33.2 | |

^a^ Country: Country: CZE = Czech Republic, DFS = Denmark, Finland and Sweden, IRL = Ireland, DEU = Germany, CHE = Switzerland.

**Table S2 List of environmental effects in the national model of each country ^a, b^.**

| **COU ^a^** | **Fixed** | | | | | | **Random** | | **Covariates** | |
| --- | --- | --- | --- | --- | --- | --- | --- | --- | --- | --- |
| CZE |  | asextwin |  | year |  |  | PE | HYS | aaca | aaca2 |
| DFS | HYS | asex | aaca | seas | twin |  | PE |  |  |  |
| IRL | HYS | asex | pariagedam |  |  |  | PE |  | agedam2 | aawg |
| DEU |  | asex | pari | month | twin |  |  | HY |  |  |
| CHE |  | asex |  | yearmonth |  | alpine | PE | HY | agedam | agedam2 |

^a^ Country: CZE = Czech Republic, DFS = Denmark, Finland and Sweden, IRL = Ireland, DEU = Germany, CHE = Switzerland.

^b^ aaca = age at calving; aaca2 = age at calving fitted as quadratic effect; aawg = age at weighting; agedam = age of the dam; agedam2 = age of the dam fitted as quadratic effect; alpine = access to alpine grazing for calves; asex = sex of the animal; asextwin = interaction between asex and twin; HY = Herd-Year; HYS = Herd-Year-Season; month = month of birth; pari = parity; pariagedam = interaction between pari and agedam; PE = maternal permanent environmental effect; seas = season; twin = twinning; year = year of birth; yearmonth = interaction between year and month.

**Table S3 Direct and maternal genetic covariances (below diagonal), genetic variances (diagonal) and genetic correlations (above diagonal) within and across countries ^a^.**

|  |  | **Direct** | | | | | **Maternal** | | | | |
| --- | --- | --- | --- | --- | --- | --- | --- | --- | --- | --- | --- |
|  |  | CZE | DFS | IRL | DEU | CHE | CZE | DFS | IRL | DEU | CHE |
| **Direct** | CZE | 686 | 0.86 | 0.82 | 0.76 | 0.83 | -0.27 | 0.00 | -0.01 | 0.00 | -0.02 |
|  | DFS | 368.13 | 269 | 0.79 | 0.89 | 0.83 | 0.00 | -0.13 | 0.00 | -0.03 | 0.00 |
|  | IRL | 452.95 | 274.16 | 450 | 0.66 | 0.75 | -0.01 | 0.00 | -0.20 | 0.01 | 0.01 |
|  | DEU | 387.46 | 286.45 | 274.70 | 383 | 0.72 | -0.01 | -0.02 | 0.00 | -0.27 | 0.00 |
|  | CHE | 424.10 | 264.64 | 309.17 | 276.30 | 380 | -0.02 | 0.00 | 0.00 | 0.00 | -0.24 |
| **Maternal** | CZE | -99.12 | 0.61 | -2.87 | -1.64 | -5.15 | 197 | 0.59 | 0.61 | 0.61 | 0.65 |
|  | DFS | 0.74 | -22.66 | -0.97 | -4.75 | -0.59 | 91.10 | 120 | 0.69 | 0.68 | 0.67 |
|  | IRL | -2.68 | 0.17 | -58.46 | 0.05 | 1.22 | 118.64 | 105.20 | 194 | 0.69 | 0.65 |
|  | DEU | -1.25 | -9.19 | 2.00 | -95.86 | 1.10 | 153.86 | 134.76 | 172.61 | 326 | 0.66 |
|  | CHE | -5.32 | 0.26 | 1.27 | 0.18 | -44.79 | 87.97 | 71.14 | 87.34 | 114.94 | 94 |

^a^ Country: CZE = Czech Republic, DFS = Denmark, Finland and Sweden, IRL = Ireland, DEU = Germany, CHE = Switzerland.

**Table S4 National genetic, environmental and residual (co)variances ^a^.**

| **COU ^b^** | **σ^2^_HY_** | **σ^2^_HYS_** | **σ^2^_PE_** | **σ^2^_dir_** | **σ^2^_mat_** | **σ_dir-mat_** | **σ^2^_res_** | **h^2^_dir_** | **h^2^_mat_** |
| --- | --- | --- | --- | --- | --- | --- | --- | --- | --- |
| CZE |  | 294 | 208 | 686 | 197 | -110.28 | 377 | 0.42 | 0.12 |
| DFS |  |  | 90 | 269 | 120 | -26.95 | 547 | 0.27 | 0.12 |
| IRL |  |  | 45 | 450 | 194 | -59.09 | 647 | 0.35 | 0.15 |
| DEU | 477 |  |  | 383 | 326 | -106.01 | 719 | 0.21 | 0.18 |
| CHE | 203 |  | 76 | 380 | 94 | -47.25 | 587 | 0.29 | 0.07 |

^a^ σ^2^ = variance, HY = Herd-Year, HYS = Herd-Year-Season, PE = maternal permanent environment, dir = direct genetic effect, mat = maternal genetic effect, σ_dir-mat_ = direct-maternal genetic covariance, res = residual, h^2^_dir_ = direct heritability, h^2^_mat_ = maternal heritability.

^b^ Country: CZE = Czech Republic, DFS = Denmark, Finland and Sweden, IRL = Ireland, DEU = Germany, CHE = Switzerland.

**Table S5 Level bias (**${\hat{\boldsymbol{\Delta}}}_{\boldsymbol{p}}$**) ^a^, dispersion bias (**${\hat{\boldsymbol{b}}}_{\boldsymbol{p}}$**) and accuracy of partial EBV (**${\hat{\boldsymbol{acc}}}_{\boldsymbol{p}}$**) of direct and maternal EBV for animals with phenotypes and no genotypes born from 2014 onwards ^b^, in each scenario ^c^ and for each country ^d^.**

|  | **Direct** | | | | **Maternal** | | | |
| --- | --- | --- | --- | --- | --- | --- | --- | --- |
| **COU** ^d^ | **PBLUP**  **_NAT_** | **ssSNPBLUP**  **_NAT_** | **PBLUP**  **_INT_** | **ssSNPBLUP**  **_INT_** | **PBLUP**  **_NAT_** | **ssSNPBLUP**  **_NAT_** | **PBLUP**  **_INT_** | **ssSNPBLUP**  **_INT_** |
| $\hat{\Delta}_{p}$ (GSD) ^a^ |  |  |  |  |  |  |  |  |
| CZE | -0.08 | -0.08 | -0.07 | -0.07 | 0.01 | 0.00 | 0.02 | 0.03 |
| DFS | -0.07 | - | -0.08 | -0.07 | -0.04 | - | -0.03 | -0.03 |
| IRL | -0.03 | -0.07 | -0.04 | -0.07 | -0.02 | 0.01 | -0.02 | 0.02 |
| DEU | 0.08 | 0.10 | 0.07 | 0.08 | -0.02 | 0.01 | -0.01 | 0.01 |
| CHE | -0.04 | -0.05 | -0.02 | -0.02 | 0.05 | 0.05 | 0.05 | 0.07 |
| Range of SE ^e^ | 0.00-0.01 | 0.00-0.01 | 0.00-0.01 | 0.00-0.01 | 0.00-0.00 | 0.00-0.00 | 0.00-0.00 | 0.00-0.00 |
| $\hat{b}_{p}$ |  |  |  |  |  |  |  |  |
| CZE | 0.77 | 0.81 | 0.74 | 0.77 | 0.75 | 0.78 | 0.90 | 0.85 |
| DFS | 0.83 | - | 0.84 | 0.87 | 0.95 | - | 0.98 | 0.98 |
| IRL | 0.94 | 0.89 | 0.96 | 0.89 | 0.90 | 0.86 | 0.88 | 0.81 |
| DEU | 0.90 | 0.90 | 0.91 | 0.92 | 0.85 | 0.86 | 0.84 | 0.84 |
| CHE | 0.95 | 0.95 | 0.95 | 0.95 | 1.04 | 1.04 | 0.98 | 1.02 |
| Range of SE ^e^ | 0.01-0.02 | 0.01-0.02 | 0.01-0.02 | 0.01-0.02 | 0.01-0.02 | 0.01-0.02 | 0.01-0.02 | 0.01-0.02 |
| $\hat{acc}_{p}$ |  |  |  |  |  |  |  |  |
| CZE | 0.27 | 0.29 | 0.29 | 0.30 | 0.16 | 0.16 | 0.18 | 0.19 |
| DFS | 0.33 | - | 0.33 | 0.36 | 0.29 | - | 0.29 | 0.30 |
| IRL | 0.20 | 0.23 | 0.22 | 0.26 | 0.15 | 0.17 | 0.15 | 0.18 |
| DEU | 0.36 | 0.39 | 0.36 | 0.39 | 0.24 | 0.24 | 0.24 | 0.24 |
| CHE | 0.45 | 0.49 | 0.46 | 0.49 | 0.22 | 0.34 | 0.23 | 0.33 |
| Range of SE ^e^ | 0.00-0.00 | 0.00-0.00 | 0.00-0.00 | 0.00-0.00 | 0.00-0.00 | 0.00-0.00 | 0.00-0.00 | 0.00-0.00 |

^a^ Level bias is expressed in genetic standard deviations (GSD). ^b^ Number of animals in each country: CZE = 5,731, DFS = 17,941, IRL = 14,638, DEU = 33,966, CHE = 9,684. ^c^ Scenario: PBLUP_NAT_ = Pedigree-based BLUP national, ssSNPBLUP_NAT_ = single-step SNP-BLUP national, PBLUP_INT_ = Pedigree-based BLUP international, ssSNPBLUP_INT_ = single-step SNP-BLUP international. ^d^ COU = Country: CZE = Czech Republic, DFS = Denmark, Finland and Sweden, IRL = Ireland, DEU = Germany, CHE = Switzerland. ^e^ Range of SE: minimum and maximum Standard Error across countries in each scenario.

**Table S6 Increases in population accuracy (**$\boldsymbol{inc\_acc}$**) of moving from each scenario to ssSNPBLUP_INT_ ^a, b^ for direct and maternal EBV for animals with phenotypes and no genotypes born from 2014 onwards ^c^ for each country ^d^.**

|  | **Direct** | | | **Maternal** | | |
| --- | --- | --- | --- | --- | --- | --- |
| **COU** ^d^ | **PBLUP**  **_NAT_** | **ssSNPBLUP**  **_NAT_** | **PBLUP**  **_INT_** | **PBLUP**  **_NAT_** | **ssSNPBLUP**  **_NAT_** | **PBLUP**  **_INT_** |
| CZE | 1.4 | 0.9 | 0.6 | 14.0 | 13.4 | 3.3 |
| DFS | 1.4 | - | 0.4 | 1.2 | - | 0.7 |
| IRL | 8.8 | 2.2 | 5.1 | 17.0 | 6.5 | 13.9 |
| DEU | 0.8 | 0.5 | 0.6 | 1.2 | 0.9 | 0.6 |
| CHE | 3.8 | 0.4 | 2.9 | 22.4 | 4.7 | 25.7 |
| Range of SE ^e^ | 0.0-0.6 | 0.0-0.0 | 0.0-0.1 | 0.0-0.6 | 0.0-0.4 | 0.0-0.7 |

^a^ Increases in population accuracies are expressed in % relative to each scenario whole EBV.

^b^ Scenario: PBLUP_NAT_ = Pedigree-based BLUP national, ssSNPBLUP_NAT_ = single-step SNP-BLUP national, PBLUP_INT_ = Pedigree-based BLUP international, ssSNPBLUP_INT_ = single-step SNP-BLUP international.

^c^ Number of animals in each country: CZE = 5,731, DFS = 17,941, IRL = 14,638, DEU = 33,966, CHE = 9,684.

^d^ COU = Country: CZE = Czech Republic, DFS = Denmark, Finland and Sweden, IRL = Ireland, DEU = Germany, CHE = Switzerland.

^e^ Range of SE: minimum and maximum Standard Error across countries in each scenario.

**Table S7 Countries sending the genotypes for Common Bulls (CB) and Common Maternal Grand-Sires (CMGS).**

| **Pair of**  **countries ^a^** | | **Sending genotype**  **for CB** | | | **Sending genotype**  **for CMGS** | | |
| --- | --- | --- | --- | --- | --- | --- | --- |
|  |  | IRL | DEU | CHE | IRL | DEU | CHE |
| CZE | DFS | 15 | 1 | 8 | 14 | 1 | 2 |
| CZE | IRL | 33 | 2 | 8 | 13 | 1 | 2 |
| CZE | DEU | 22 | 4 | 12 | 15 | 13 | 2 |
| CZE | CHE | 9 | 1 | 10 | 7 | 2 | 2 |
| DFS | IRL | 22 | 1 | 9 | 12 | 0 | 3 |
| DFS | DEU | 21 | 3 | 13 | 16 | 2 | 3 |
| DFS | CHE | 8 | 0 | 13 | 9 | 1 | 3 |
| IRL | DEU | 39 | 2 | 8 | 18 | 1 | 3 |
| IRL | CHE | 10 | 1 | 11 | 9 | 1 | 2 |
| DEU | CHE | 13 | 4 | 30 | 9 | 15 | 12 |

**^a^** Country: CZE = Czech Republic, DFS = Denmark, Finland and Sweden, IRL = Ireland, DEU = Germany, CHE = Switzerland.

**Table S8 Level bias (**${\hat{\boldsymbol{\Delta}}}_{\boldsymbol{p}}$**) ^a^, dispersion bias (**${\hat{\boldsymbol{b}}}_{\boldsymbol{p}}$**) and accuracy of partial EBV (**${\hat{\boldsymbol{acc}}}_{\boldsymbol{p}}$**) of direct and maternal EBV for the focal group ^b^, in each scenario ^c^ and for each country ^d^. Standard errors reported within parenthesis.**

|  | **Direct** | | | | **Maternal** | | | |
| --- | --- | --- | --- | --- | --- | --- | --- | --- |
| **COU** ^d^ | **PBLUP**  **_NAT_** | **ssSNPBLUP**  **_NAT_** | **PBLUP**  **_INT_** | **ssSNPBLUP**  **_INT_** | **PBLUP**  **_NAT_** | **ssSNPBLUP**  **_NAT_** | **PBLUP**  **_INT_** | **ssSNPBLUP**  **_INT_** |
| $\hat{\Delta}_{p}$ (GSD) ^a^ |  |  |  |  |  |  |  |  |
| CZE | -0.25 (0.01) | -0.23 (0.01) | -0.23 (0.01) | -0.22 (0.01) | -0.01 (0.01) | -0.03 (0.01) | 0.00 (0.01) | 0.01 (0.01) |
| IRL | -0.08 (0.01) | -0.10 (0.01) | -0.10 (0.01) | -0.10 (0.01) | -0.02 (0.00) | 0.01 (0.00) | -0.01 (0.00) | 0.02 (0.00) |
| DEU | -0.19 (0.02) | -0.10 (0.02) | -0.21 (0.02) | -0.15 (0.02) | -0.06 (0.01) | -0.02 (0.01) | -0.05 (0.01) | -0.02 (0.01) |
| CHE | -0.28 (0.01) | -0.27 (0.01) | -0.23 (0.01) | -0.21 (0.01) | 0.04 (0.01) | 0.03 (0.01) | 0.04 (0.01) | 0.06 (0.01) |
| $\hat{b}_{p}$ |  |  |  |  |  |  |  |  |
| CZE | 0.72 (0.05) | 0.76 (0.05) | 0.65 (0.05) | 0.79 (0.03) | 1.04 (0.05) | 0.94 (0.05) | 1.06 (0.05) | 0.96 (0.04) |
| IRL | 0.96 (0.02) | 0.87 (0.02) | 1.00 (0.02) | 0.87 (0.02) | 0.92 (0.02) | 0.87 (0.02) | 0.91 (0.02) | 0.85 (0.02) |
| DEU | 0.79 (0.07) | 0.85 (0.06) | 0.77 (0.06) | 0.82 (0.04) | 0.78 (0.07) | 0.79 (0.07) | 0.79 (0.07) | 0.79 (0.05) |
| CHE | 0.80 (0.03) | 0.79 (0.03) | 0.80 (0.03) | 0.82 (0.03) | 1.06 (0.04) | 0.98 (0.04) | 0.99 (0.03) | 0.93 (0.03) |
| $\hat{acc}_{p}$ |  |  |  |  |  |  |  |  |
| CZE | 0.23 (0.01) | 0.25 (0.01) | 0.25 (0.01) | 0.35 (0.01) | 0.17 (0.01) | 0.17 (0.01) | 0.19 (0.01) | 0.23 (0.01) |
| IRL | 0.23 (0.00) | 0.29 (0.00) | 0.26 (0.00) | 0.35 (0.01) | 0.17 (0.00) | 0.22 (0.00) | 0.18 (0.00) | 0.24 (0.00) |
| DEU | 0.26 (0.02) | 0.31 (0.02) | 0.27 (0.02) | 0.35 (0.02) | 0.18 (0.01) | 0.20 (0.01) | 0.18 (0.01) | 0.23 (0.01) |
| CHE | 0.34 (0.01) | 0.38 (0.01) | 0.35 (0.01) | 0.40 (0.01) | 0.22 (0.01) | 0.27 (0.01) | 0.24 (0.01) | 0.29 (0.01) |

^a^ Level bias is expressed in genetic standard deviations (GSD).

^b^ focal group: animals with phenotypes and genotypes born from 2014 onwards.

^c^ Scenario: PBLUP_NAT_ = Pedigree-based BLUP national, ssSNPBLUP_NAT_ = single-step SNP-BLUP national, PBLUP_INT_ = Pedigree-based BLUP international, ssSNPBLUP_INT_ = single-step SNP-BLUP international.

^d^ COU = Country: CZE = Czech Republic, DFS = Denmark, Finland and Sweden, IRL = Ireland, DEU = Germany, CHE = Switzerland.
