# Supplementary material for: International single-step SNPBLUP beef cattle evaluations for Limousin weaning weight
Source: Genet Sel Evol. 2022 Sep 4;54:57. doi: 10.1186/s12711-022-00748-0 (PMC9441073; doi:10.1186/s12711-022-00748-0)
Supplement: Supplementary file 2 — Additional file 2: File S1. Findhap instruction file. File S2. International pedigree-based BLUP MiXBLUP instruction file. File S3. International single-step SNPBLUP MiXBLUP instruction file. [file 12711_2022_748_MOESM2_ESM.docx]

# Additional file 2

**File S1. Findhap instruction file.**

4 0 600 75 3 50000 1 5 0 1 .004

iters Xchrom maxlen minlen steps maxhap hapout genout damout listout errrate

**File S2. International pedigree-based BLUP MiXBLUP instruction file.**

TITLE International pedigree-based BLUP

DATAFILE data.dat !MISSING 0

ANIM I

DAM I

DAMPE I

R1CZE I

F1CZE I

F2CZE I

F1DFS I

F2DFS I

F3DFS I

F4DFS I

F5DFS I

F1IRL I

F2IRL I

F3IRL I

R1DEU I

F1DEU I

F2DEU I

F3DEU I

F4DEU I

R1CHE I

F1CHE I

F2CHE I

F3CHE I

HERD I

X1CZE R

X2CZE R

X1IRL R

X2IRL R

X1CHE R

X2CHE R

YCZE T

YDFS T

YIRL T

YDEU T

YCHE T

POPCODE A

PEDFILE pedigree.ped !CalcInbr

ANIM I

SIRE I

DAM I

HERD I

PARFILE parfile.par

MODEL

YCZE ~ X1CZE X2CZE F1CZE F2CZE !random R1CZE DAMPE G(ANIM, DAM)

YDFS ~ F1DFS F2DFS F3DFS F4DFS F5DFS !random DAMPE G(ANIM, DAM)

YIRL ~ X1IRL X2IRL F1IRL F2IRL F3IRL !random DAMPE G(ANIM, DAM)

YDEU ~ F1DEU F2DEU F3DEU F4DEU !random R1DEU G(ANIM, DAM)

YCHE ~ X1CHE X2CHE F1CHE F2CHE F3CHE !random R1CHE DAMPE G(ANIM, DAM)

SOLVING

!STOPCRIT 1.0E-05

!numproc 3

!KEEPTMP

**File S3. International single-step SNPBLUP MiXBLUP instruction file.**

TITLE International single-step SNPBLUP

DATAFILE data.dat !MISSING 0

ANIM I

DAM I

DAMPE I

R1CZE I

F1CZE I

F2CZE I

F1DFS I

F2DFS I

F3DFS I

F4DFS I

F5DFS I

F1IRL I

F2IRL I

F3IRL I

R1DEU I

F1DEU I

F2DEU I

F3DEU I

F4DEU I

R1CHE I

F1CHE I

F2CHE I

F3CHE I

HERD I

X1CZE R

X2CZE R

X1IRL R

X2IRL R

X1CHE R

X2CHE R

YCZE T

YDFS T

YIRL T

YDEU T

YCHE T

POPCODE A

PEDFILE pedigree.ped !CalcInbr !makeJcov

ANIM I

SIRE I

DAM I

HERD I

PARFILE parfile.par

SNPFILE !NoCheck !CalcSNPvar !PREDICT !PLINK

ANIM I

SNP01 Plink_geno.bed !REGTYPE r

REGFILE

ANIM I

REG01 !REGTYPE F !Jcov

MODEL

YCZE ~ X1CZE X2CZE F1CZE F2CZE hpReg(01,ANIM) hpReg(01,DAM) !random R1CZE DAMPE hpSNP(01,ANIM) hpSNP(01,DAM) G(ANIM, DAM)

YDFS ~ F1DFS F2DFS F3DFS F4DFS F5DFS hpReg(01,ANIM) hpReg(01,DAM) !random DAMPE hpSNP(01,ANIM) hpSNP(01,DAM) G(ANIM, DAM)

YIRL ~ X1IRL X2IRL F1IRL F2IRL F3IRL hpReg(01,ANIM) hpReg(01,DAM) !random DAMPE hpSNP(01,ANIM) hpSNP(01,DAM) G(ANIM, DAM)

YDEU ~ F1DEU F2DEU F3DEU F4DEU hpReg(01,ANIM) hpReg(01,DAM) !random R1DEU hpSNP(01,ANIM) hpSNP(01,DAM) G(ANIM, DAM)

YCHE ~ X1CHE X2CHE F1CHE F2CHE F3CHE hpReg(01,ANIM) hpReg(01,DAM) !random R1CHE DAMPE hpSNP(01,ANIM) hpSNP(01,DAM) G(ANIM, DAM)

SOLVING

!STOPCRIT 1.0E-05

!hpblup

!hpSNPmodel liu

!numproc 3

!KEEPTMP
