# Supplementary material for: International single-step SNPBLUP beef cattle evaluations for Limousin weaning weight
Source: Genet Sel Evol. 2022 Sep 4;54:57. doi: 10.1186/s12711-022-00748-0 (PMC9441073; doi:10.1186/s12711-022-00748-0)
Supplement: Supplementary file 3 — Additional file 3: Figure S1. Plot of pedigree-based (x-axis) and genomic-based (y-axis) relationships between genotyped animals. The red dots indicate the relationships of the 41 genotypes removed due to pedigree incompatibilities. Figure S2. Differences in genetic trends between ssSNPBLUPINT and PBLUPINT per country for sires with at least 10 recorded offspring in the country (only birth years with at least 5 sires are included). [file 12711_2022_748_MOESM3_ESM.docx]

# Additional file 3

**Figure S1 Plot of pedigree-based (x-axis) and genomic-based (y-axis) relationships between genotyped animals**. The red dots indicate the relationships of the 41 genotypes removed due to pedigree incompatibilities.


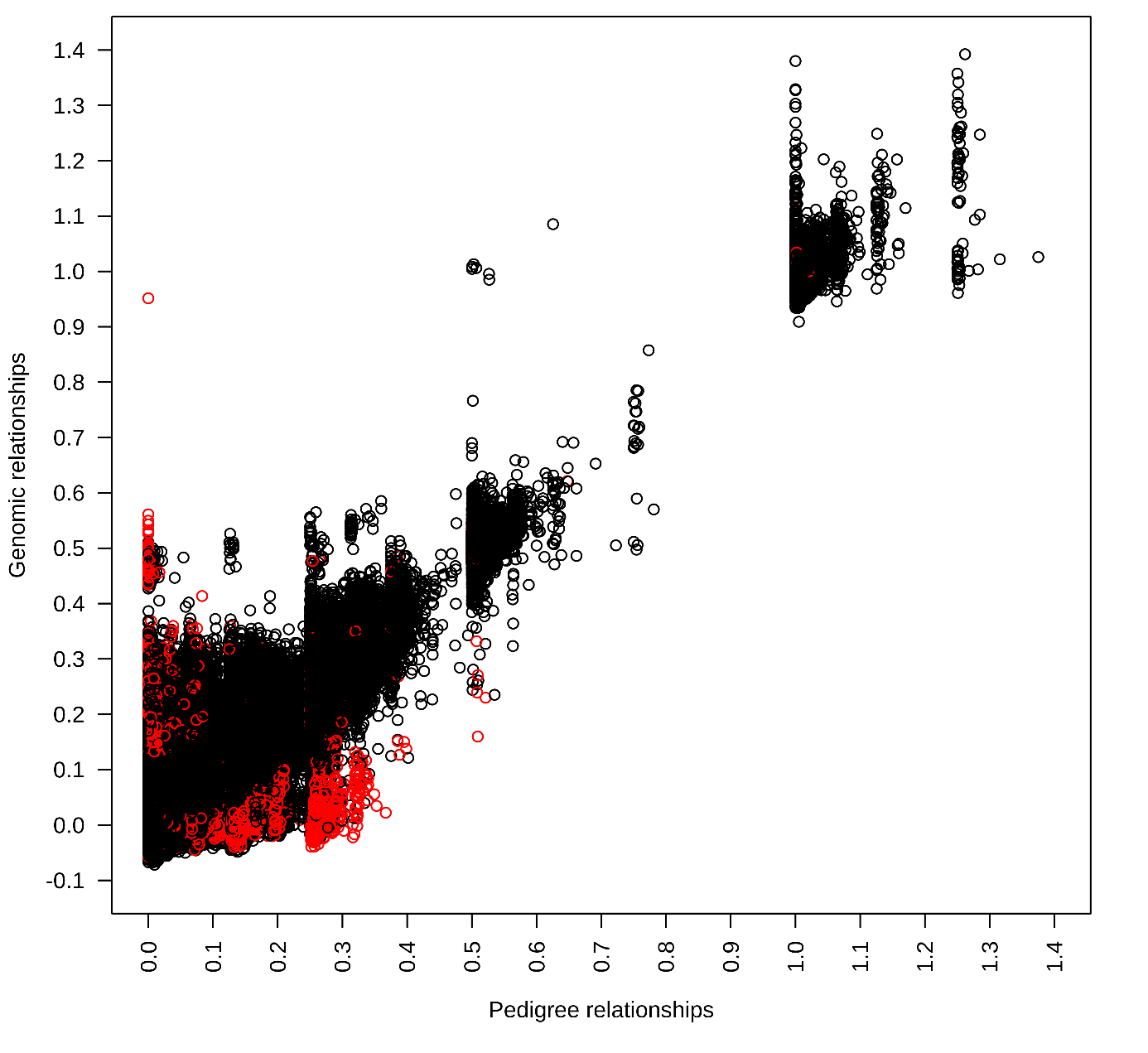


**Figure S2** **Differences in genetic trends ^a^ between ssSNPBLUP_INT_ and PBLUP_INT_ per country ^b^ for sires with at least 10 recorded offspring in the country (only birth years with at least 5 sires are included).**


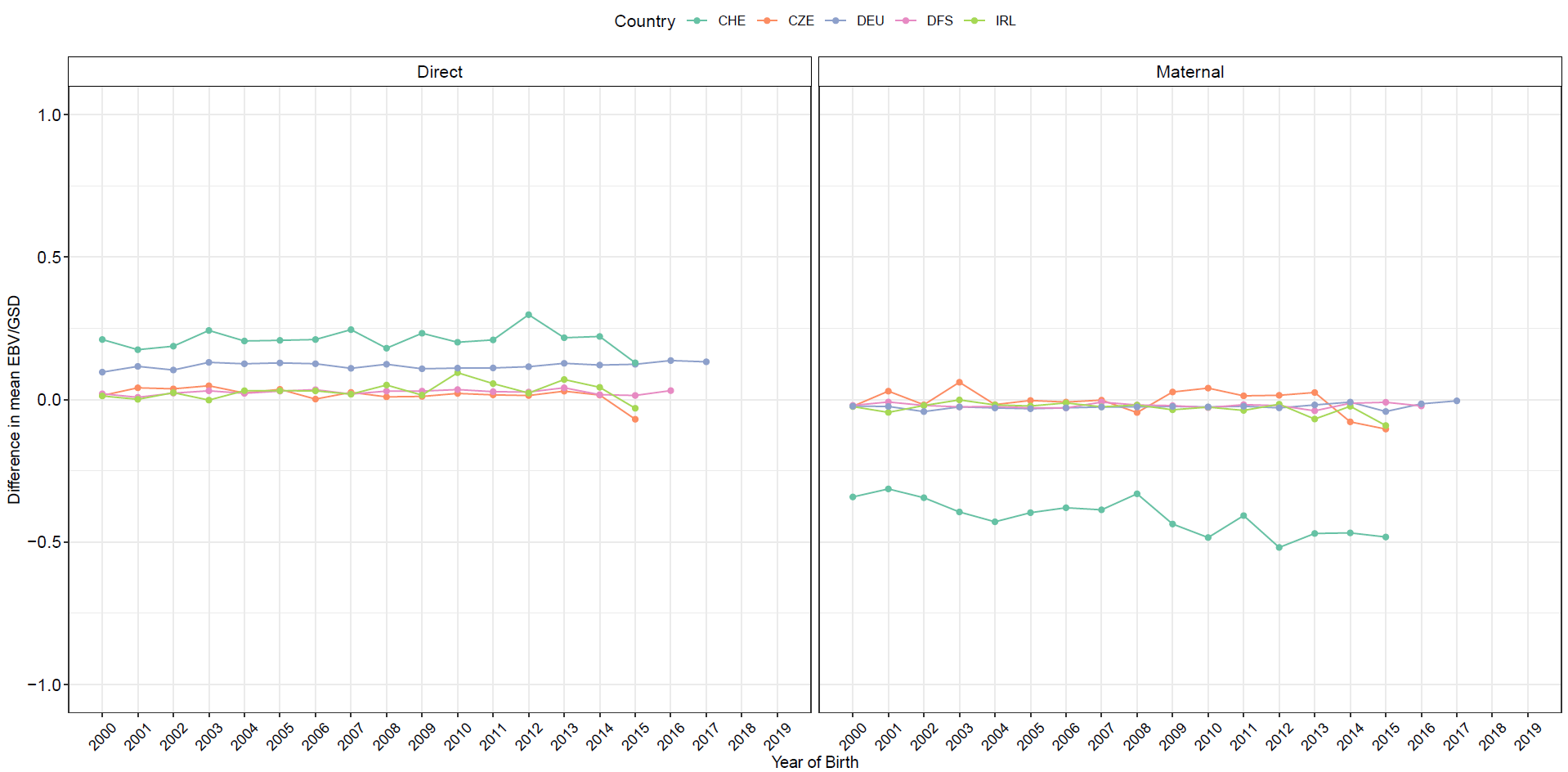


^a^ Differences between the mean EBV in ssSNPBLUP_INT_ and the mean EBV in PBLUP_INT_ for the period 2000-2019 in each country expressed in genetic standard deviations (GSD).

^b^ CZE = Czech Republic, DFS = Denmark, Finland and Sweden, IRL = Ireland, DEU = Germany, CHE = Switzerland.
