# Supplementary material for: International single-step SNPBLUP beef cattle evaluations for Limousin weaning weight
Source: Genet Sel Evol. 2022 Sep 4;54:57. doi: 10.1186/s12711-022-00748-0 (PMC9441073; doi:10.1186/s12711-022-00748-0)
Supplement: Supplementary file 4 — Additional file 4: Figure S3. Direct and maternal EBV realized Mendelian sampling (RMS) trends in each countrya expressed in genetic standard deviation (GSD) for genotyped and non-genotyped animalsb computed with pedigree-based BLUP international (PBLUPINT) and single-step SNP-BLUP international (ssSNPBLUPINT) models. aCZE Czech Republic, DFS Denmark, Finland and Sweden, IRL Ireland, DEU Germany, CHE Switzerland. bGenotyped animals: animals with genotype that appear in the pseudo-national pedigree (with or without phenotype in the country). Non-genotyped animals: animals that appear in the pseudo-national pedigree without genotype and with phenotype in the country. [file 12711_2022_748_MOESM4_ESM.pdf]

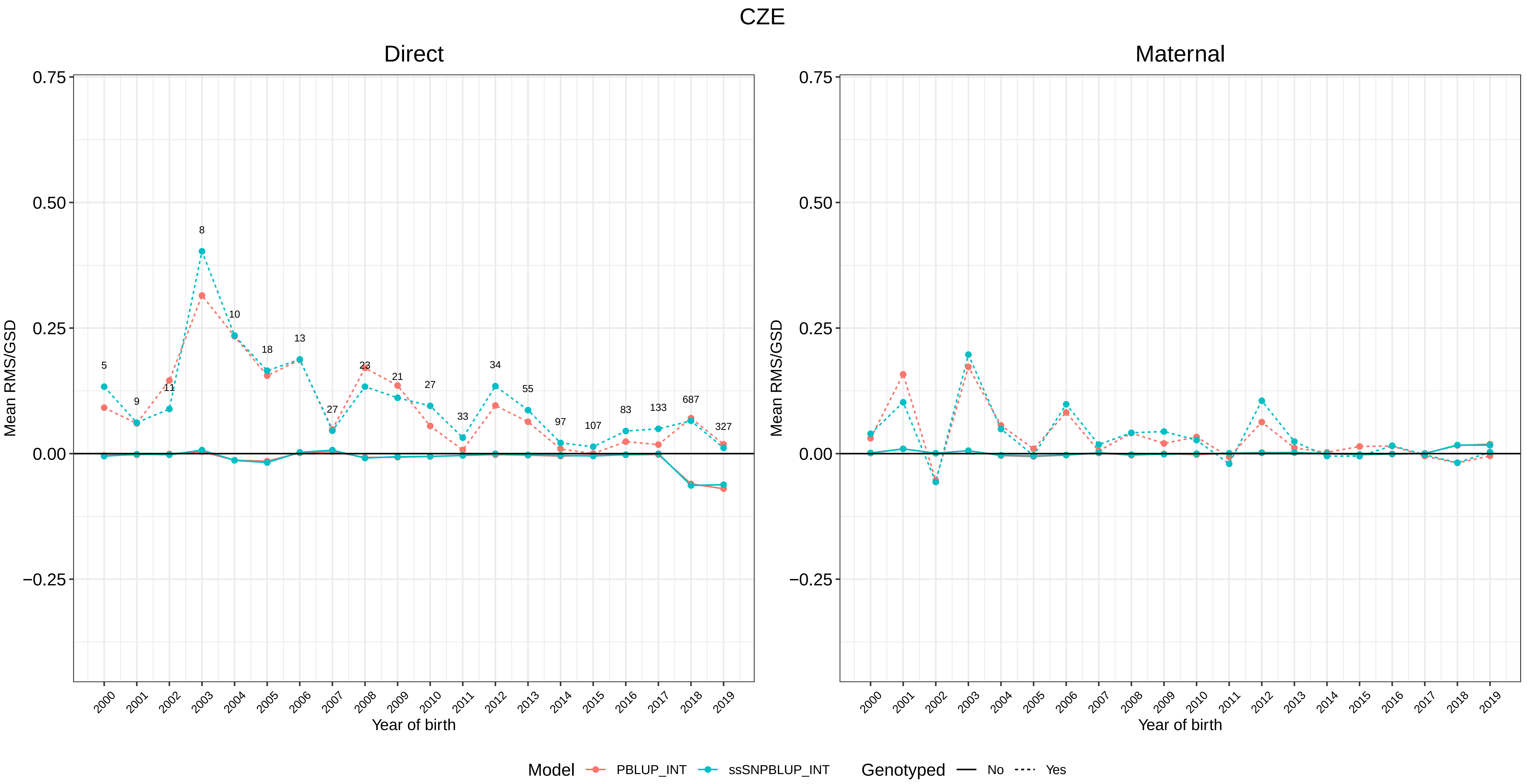

The numbers reported in the direct panel are the number of genotyped animals in that year (same numbers for the maternal panel).

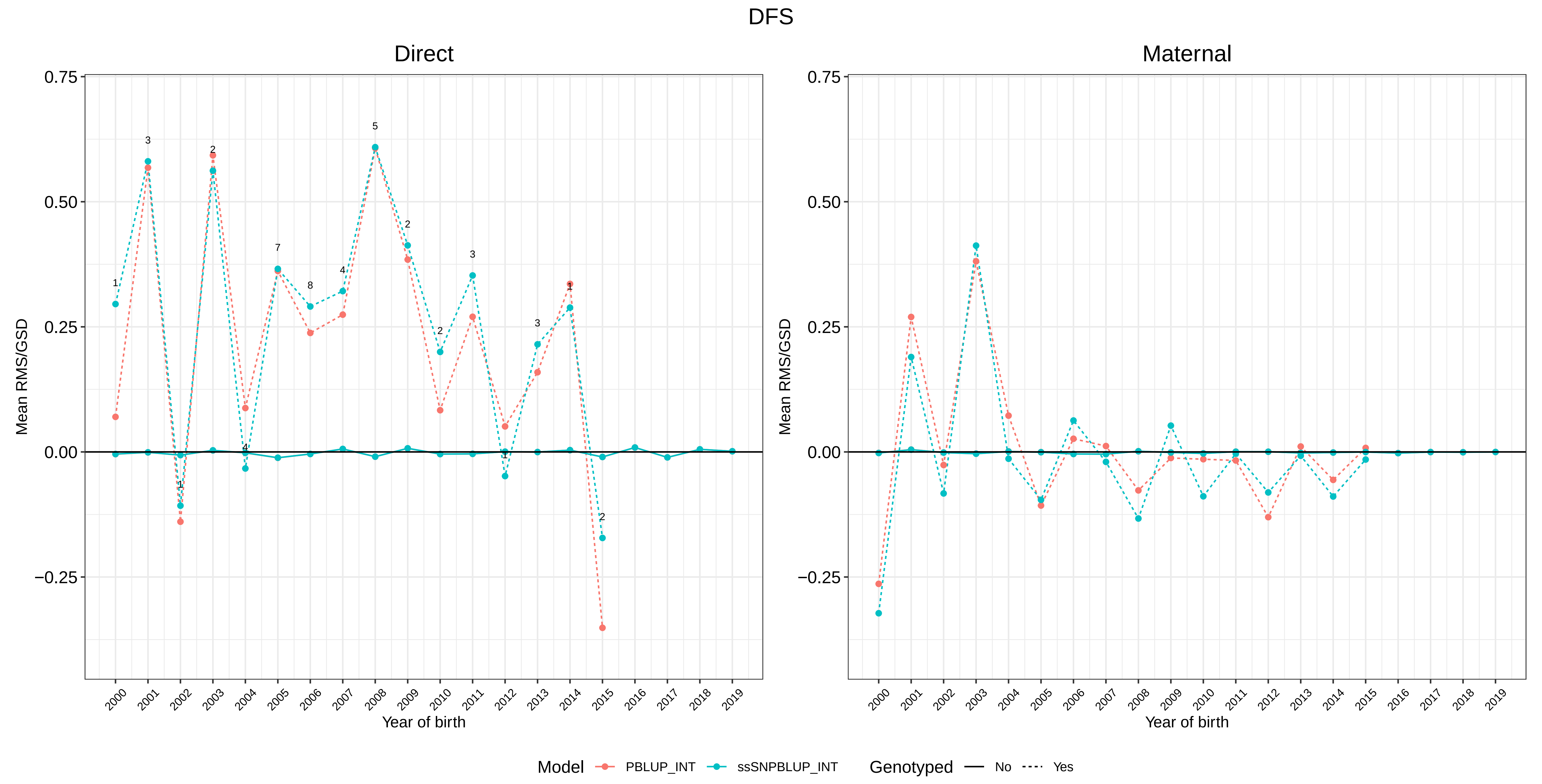

The numbers reported in the direct panel are the number of genotyped animals in that year (same numbers for the maternal panel).

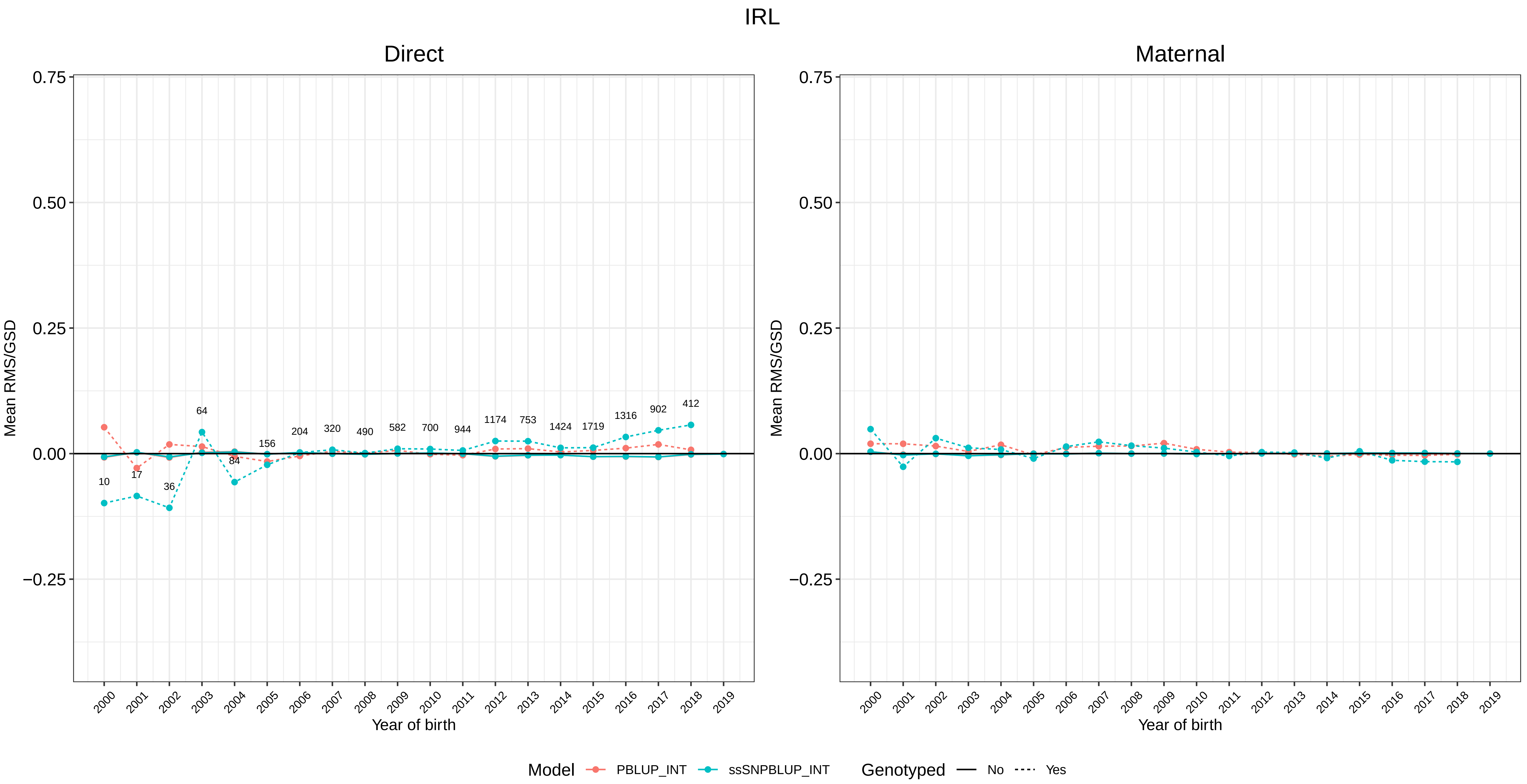

The numbers reported in the direct panel are the number of genotyped animals in that year (same numbers for the maternal panel).

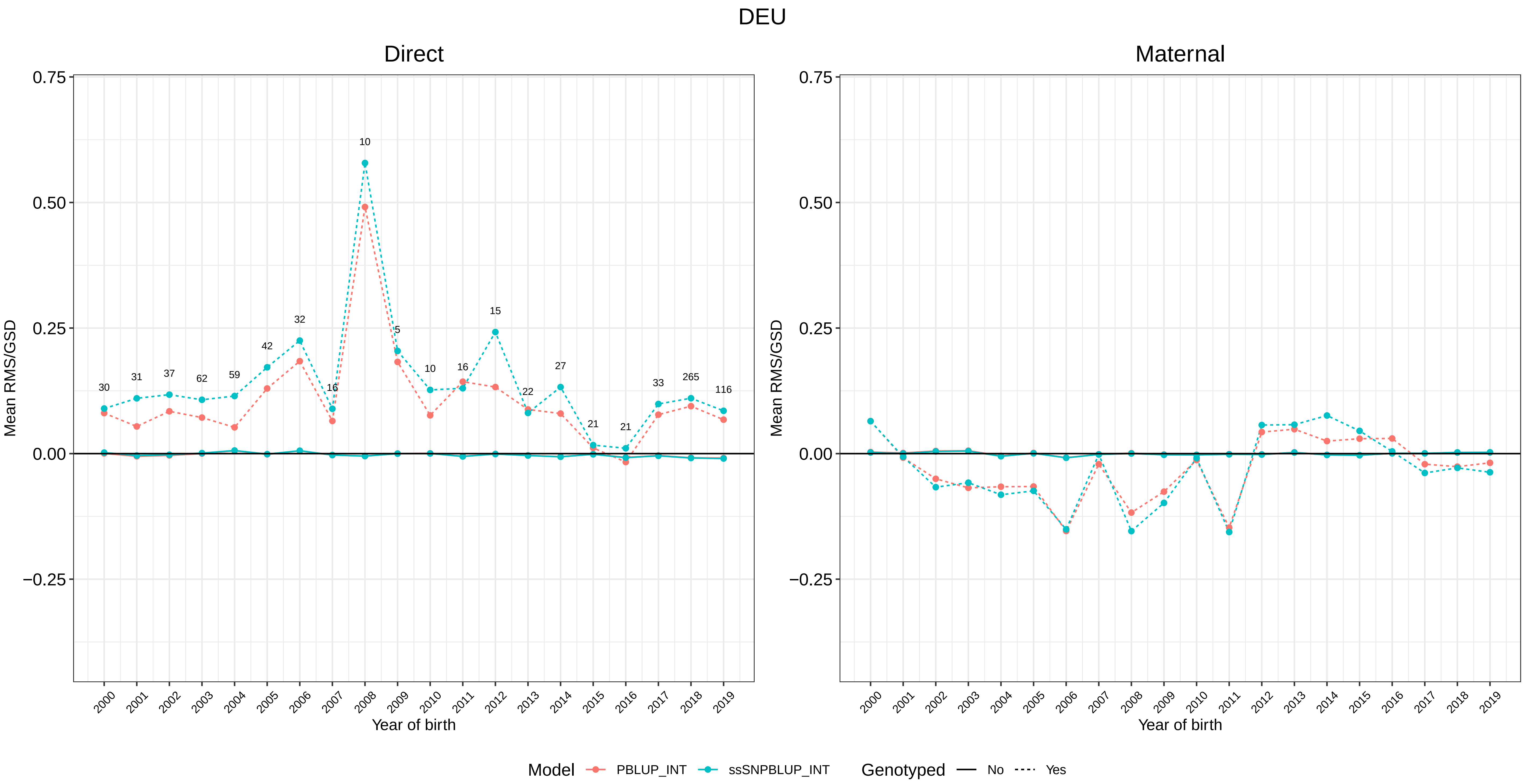

The numbers reported in the direct panel are the number of genotyped animals in that year (same numbers for the maternal panel).

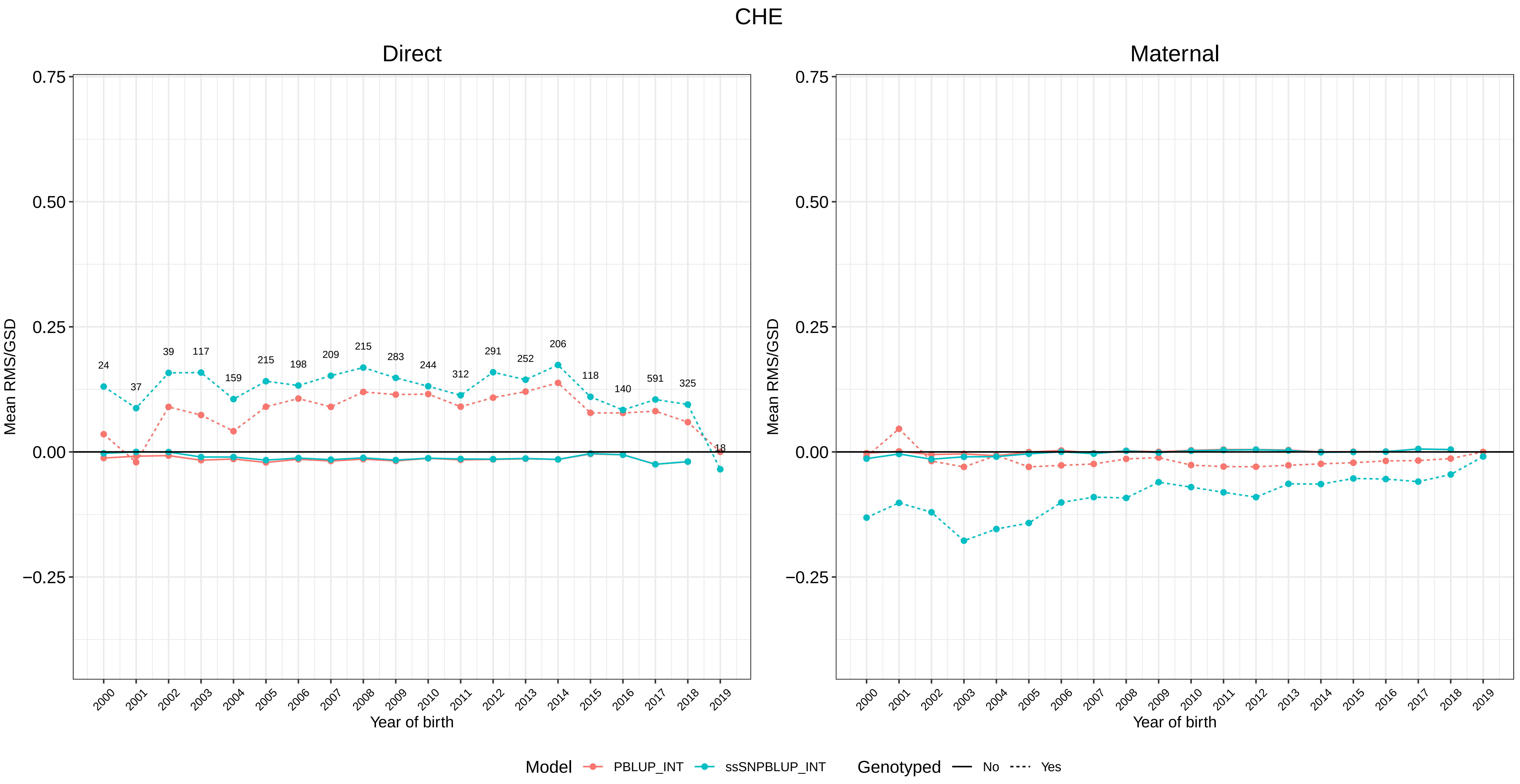

The numbers reported in the direct panel are the number of genotyped animals in that year (same numbers for the maternal panel).
